# Soret–Dufour impact on a three-dimensional Casson nanofluid flow with dust particles and variable characteristics in a permeable media

**DOI:** 10.1038/s41598-021-93797-2

**Published:** 2021-07-15

**Authors:** Naila Shaheen, Muhammad Ramzan, Ahmed Alshehri, Zahir Shah, Poom Kumam

**Affiliations:** 1 Department of Computer Science, Bahria University, Islamabad, 44000 Pakistan; 2 Department of Mathematics, Faculty of Sciences, King Abdulaziz University, Jeddah, 21589 Saudi Arabia; 3 Department of Mathematical Sciences, University of Lakki Marwat, Lakki Marwat, Khyber Pakhtunkhwa 28420 Pakistan; 4 Center of Excellence in Theoretical and Computational Science (TaCS-CoE), Faculty of Science, King Mongkut’s University of Technology Thonburi (KMUTT), 126 Pracha Uthit Rd., Bang Mod, Thung Khru, Bangkok, 10140 Thailand; 5 Fixed Point Research Laboratory, Fixed Point Theory and Applications Research Group, Center of Excellence in Theoretical and Computational Science (TaCS-CoE), Faculty of Science, King Mongkut’s University of Technology Thonburi (KMUTT), 126 Pracha Uthit Rd., Bang Mod, Thung Khru, Bangkok, 10140 Thailand; 6 Department of Medical Research, China Medical University Hospital, China Medical University, Taichung, 40402 Taiwan

**Keywords:** Mechanical engineering, Software

## Abstract

In this study, the effects of variable characteristics are analyzed on a three-dimensional (3D) dusty Casson nanofluid flow past a deformable bidirectional surface amalgamated with chemical reaction and Arrhenius activation energy. The surface is deformable in the direction of the *x-*axis and *y-*axis. The motion of the flow is induced due to the deformation of the surface. The impression of Soret and Dufour's effects boost the transmission of heat and mass. The flow is analyzed numerically with the combined impacts of thermal radiation, momentum slip, and convective heat condition. A numerical solution for the system of the differential equations is attained by employing the bvp4c function in MATLAB. The dimensionless parameters are graphically illustrated and discussed for the involved profiles. It is perceived that on escalating the Casson fluid and porosity parameters, the velocity field declines for fluid-particle suspension. Also, for augmented activation energy and Soret number, the concentration field enhances. An opposite behavior is noticed in the thermal field for fluctuation in fluid-particle interaction parameters for fluid and dust phase. Drag force coefficient increases on escalating porosity parameter and Hartmann number. On amplifying the radiation parameter heat and mass flux augments. A comparative analysis of the present investigation with an already published work is also added to substantiate the envisioned problem.

## Introduction

The dusty fluid is formed with the amalgamation of dust granules with base fluid. Researchers have immensely emphasized fluid-particle suspension past an elongated surface as it has enormous applications in industry, engineering, and in the field of medicine such as power technology, cooling of nuclear reactors, power plant piping, retrieval of crude oil, sedimentation process, wastewater treatment, the formation of raindrops, emission of smoke from vehicles and environmental pollution. Hady and Mahdy^1^ presented the convective flow of an electrically conducting dusty Micropolar fluid in a porous chamber with convective heat conditions. It is observed here that the temperature field of dusty granules elevates on incrementing the fluid-particle interaction parameter. A numerical solution for time-independent two-phase Jeffery fluid flow is presented by Zokri et al.^2^ past a shrinking surface. The flow is incorporated with the effect of suction and Newtonian heating. It is found that the velocity of dusty flow upsurges on increasing the fluid-particle interaction parameter, whereas, for fluid flow, an opposite behavior is observed. Bio convective dusty nano liquid flow is numerically probed by Dey et al.^3^ over a vertical elongated surface. It is reported that the concentration of microorganisms augments for rising values of the Brownian motion parameter. Bibi et al.^4^ numerically inspected time-dependent nonlinear radiative two-phase pseudoplastic fluid flow over an elongated surface. It is perceived that enhancing the nonlinear thermal radiation parameter temperature for both phases escalates. Subsequently, exploration in this regard with different physical aspects can be seen in Refs.^5–9^.

In the fluid flow, two mechanisms are involved in the conduction of heat. First, when the collision amid the molecules increases. Second, thermal conductivity plays a key role in escalating the random movement among the molecules. Thermal conductivity has significant applications in steam generators, electrolytes, concrete heating, and laminating. The characteristics of temperature-dependent thermal conductivity assimilated with mass diffusion on a radiative Casson fluid embedded in a porous medium past an elongated surface are analytically exhibited by Sohail et al.^10^. The findings disclosed that on escalating the Hartmann number and thermal radiation parameter, thermal field upsurges. The time-dependent flow of Pseudoplastic fluid past an extendable surface incorporated with homogeneous heterogeneous (h–h) reaction is numerically scrutinized by Hamid^11^. In this study, a substantial upsurge is noticed in the temperature field on augmenting the variable thermal conductivity. The features of the heat flux model on a time-independent 3D flow of non-Newtonian fluid are studied by Ramadevi et al.^12^ with irregular heat source/sink past an elongated surface. It is noticed that the coefficient of mass transfer upsurges for rising values of the chemical reaction and stretching ratio parameter. Lu et al.^13^ analytically explored the outcome of temperature-dependent thermal conductivity combined with nonlinear thermal radiation on a magnetohydrodynamic Oldroyd-B nanofluid flow over a bidirectional elongated sheet with robin conditions. Further analysis of temperature-dependent thermal conductivity is mentioned in Refs.^10,14–22^.

The Soret–Dufour factor plays a key role in the transmission of heat and mass on a moving fluid. It has a vital role in several applications which include the design of nuclear reactors, geothermal energy, groundwater pollutant migration, oil reservoirs, isotopes separation, manufacture of rubber and plastic sheets, the mixture of gases, compact heat insulation exchanger, and nuclear waste disposal. Radiative flux with Soret–Dufour effect on a Darcy Forchheimer (DF) nano liquid flow past a linear elongated sheet is illustrated by Rasool et al.^23^. It is noticed that for growing values of Soret number, solutal field augments. Similar behavior is observed in the thermal field for the Dufour number. Using the Boungirono model Prasad et al.^24^ explored the mechanism of Soret–Dufour effect on a 3D convective Oldroyd-B fluid flow past a deforming surface with velocity slip and convective heat condition. It is reported that fluid velocity upsurges on incrementing the Deborah number. On a Micropolar nanofluid flow, Ibrahim et al.^25^ investigated the impact of the Soret and Dufour factor with multiple slip conditions past a bidirectional surface. The characteristic of heat and mass transfer on a mixed convective Jeffery fluid flow over a bidirectional stretchable sheet amalgamated with Soret–Dufour effect and chemical reaction is examined by Iftikhar et al.^26^. Significant researches in this direction are mentioned in Refs.^27–38^.

Researchers have manifested concern about fluid flow across the permeable surface. The flow through the porous chamber is very common and has widespread applications in industries, petroleum, chemical engineering for instance crude oil extraction, storage of nuclear waste material, movement of oil and water across the oil reservoir, heat exchangers, drying process, MHD generators, seepage of water in river beds, filtration, and water purification process. On a radiative Maxwell nanofluid flow, Jawad et al.^39^ analytically investigated the impact of the Soret–Dufour factor on a nonlinear elongated porous surface. Variable characteristics of Newtonian fluid with thermal radiation on a deforming sheet immersed in a porous medium are explored by Megahed et al.^40^. It is reported that on enhancing the viscosity and magnetic parameter, heat flux diminishes. Irfan et al.^41^ reported the influence of chemical reaction and internal heat generation/absorption on a radiative bio-nanofluid flow past a deforming surface with stagnation point flow in a porous chamber. On a time-dependent viscous fluid flow, Rosali et al.^42^ investigated transmission of heat amalgamated with stagnation point flow past a deforming surface with porosity effect. Substantial research past a permeable deformable surface with several physical aspects is cited in Refs.^43–57^.

The aforementioned studies revealed that a good number of studies may be quoted that discuss the nanofluid flow with Soret–Dufour effects past an extended surface. However, the 3D two-phase Casson nanofluid flow amalgamated with dust particles and variable thermal conductivity amalgamated with mass diffusion is still scarce. The impression of the Soret and Dufour effect boosts the transmission of heat and mass. The flow is analyzed numerically with the combined impact of thermal radiation, chemical reaction with activation energy, momentum slip, and convective heat condition. The mathematical model is deciphered through MATLAB software bvp4c. The outcome of numerous parameters is examined via tabular and graphical illustrations. The novelty of the presented mathematical model is illustrated in Table 1 by comparing it with the published studies.

**Table 1:** Literature survey for the originality of the presented mode with contemporary published studies.

Authors	Soret Dufour effect	3D flow		Dusty fluid	Temperature-dependent thermal conductivity	Thermal radiation	Variable molecular diffusivity	Porous medium	Activation energy
Bibi et al.^4^	No	Yes		Yes	No	Yes	No	No	No
Sohail et al.^10^	No	Yes		No	Yes	Yes	Yes	No	No
Ramadevi et al.^12^	No	Yes		No	Yes	No	No	No	No
Joshi et al.^58^	No	Yes		No	No	No	No	No	No
Ramzan et al.^59^	No	Yes		No	No	No	No	No	Yes
Reddy et al.^60^	Yes	Yes		No	No	Yes	No	No	No
Waqas et al.^61^	No	Yes		No	Yes	Yes	No	No	Yes
Present	Yes	Yes		Yes	Yes	Yes	Yes	Yes	Yes

## Formation of the mathematical model

**Figure 1:**
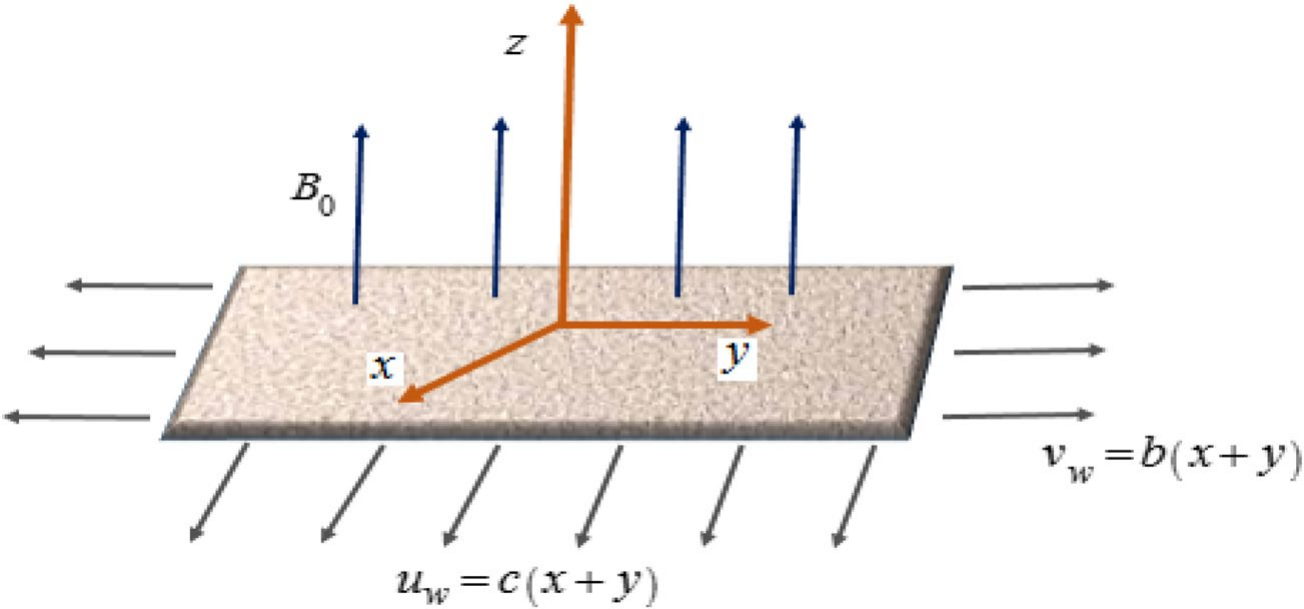
Flow representation of the model.

An incompressible, time-independent 3D magnetohydrodynamic dusty radiative Casson nano liquid flow is examined past a deformable surface embedded in a porous medium. The nano-liquid model describes the attributes of Brownian motion and thermophoresis. For the geometry of the problem, a Cartesian coordinate system is considered in such a manner that *z*-axis is perpendicular to $xy - {\text{plane}}{\text{.}}$ The flow of the subject nanofluid is at the surface $z > 0$ which is generated by a linear bidirectional stretchable surface. The surface is deformable with velocities $u_{w}  = \left( {x + y} \right)c$ and $v_{w}  = \left( {x + y} \right)b$ in the direction of *x*- and *y*-axis (Fig. 1). Transfer of heat and mass is enhanced with temperature-dependent thermal conductivity, variable molecular diffusivity incorporated with Soret and Dufour effect. Moreover, the impression of chemical reaction with activation energy and convective heat condition is also analyzed.

For an incompressible flow of Casson fluid extra stress tensor is delineated as^15^:

1 $$ \tau _{{ij}}  = \left\{ \begin{gathered}   \left( {\mu _{c}  + \frac{{S_{y} }}{{\left( {2\tilde{\pi }} \right)^{{0.5}} }}} \right)2\tilde{\gamma }_{{ij}} ,\quad if\,{\text{ }}\tilde{\pi } > \tilde{\pi }_{c}  \hfill \\   \left( {\mu _{c}  + \frac{{S_{y} }}{{\left( {2\tilde{\pi }_{c} } \right)^{{0.5}} }}} \right)2\tilde{\gamma }_{{ij}} ,\quad if\,{\text{ }}\tilde{\pi } < \tilde{\pi }_{c} , \hfill \\  \end{gathered}  \right. $$

where

2 $$ \begin{array}{c}   S_{y} \,{\text{is }}\,{\text{the }}\,{\text{yield}}\,{\text{ stress }}\,{\text{of }}\,{\text{the }}\,{\text{fluid}} \hfill \\   \tilde{\pi } = \tilde{\gamma }_{{ij}} \tilde{\gamma }_{{ij}} \,{\text{is}}\,{\text{ the}}\,{\text{ product}}\,{\text{ of}}\,{\text{ the}}\,{\text{ components }}\,{\text{of }}\,{\text{deformation }}\,{\text{rate}} \hfill \\   \tilde{\gamma }_{{ij}}  = \frac{1}{2}\left( {{v_{xi} } + {v_{yj} } } \right)\,{\text{is}}\,{\text{ the}}\,{\text{ rate}}\,{\text{ of}}\,{\text{ the}}\,{\text{ strain }}\,{\text{tensor}} \hfill \\   \tilde{\pi }_{c} \,{\text{is}}\,{\text{ the}}\,{\text{ critical}}\,{\text{ value}}\,{\text{ of}}\,{\text{ the}}\,{\text{ product}}\,{\text{ of}}\,{\text{ the}}\,{\text{ components}}\,{\text{ of }}\,{\text{deformation}}\,{\text{ rate }}\,{\text{tensor}}. \hfill \\  \end{array} $$

The equations governing the mathematical model with fluid particle suspension^1,23,31,62,63^ are:

For fluid flow:

3 $$ \tilde{u}_{x}  + \tilde{v}_{y} + \tilde{w}_{z} = 0, $$

4 $$ \tilde{u}\tilde{u}_x + \tilde{v}\tilde{u}_y + \tilde{w}\tilde{u}_z = \nu \left( {1 + \frac{1}{\beta }} \right)\tilde{u}_{zz} - \frac{{\sigma _{1} B_{0}^{2} }}{\rho }\tilde{u} - \frac{\nu }{{K^{*} }}\tilde{u} + \frac{{KN}}{\rho }\left( {\tilde{u}_{p}  - \tilde{u}} \right), $$

5 $$ \tilde{u}\tilde{v}_x + \tilde{v}\tilde{v}_y + \tilde{w}\tilde{v}_z = \nu \left( {1 + \frac{1}{\beta }} \right)\tilde{v}_{zz} - \frac{{\sigma _{1} B_{0}^{2} }}{\rho }\tilde{v} - \frac{\nu }{{K^{*} }}\tilde{v} + \frac{{KN}}{\rho }\left( {\tilde{v}_{p}  - \tilde{v}} \right), $$

6 $$ \begin{aligned}   \tilde{u}\tilde{T}_x + \tilde{v} \tilde{T}_y + \tilde{w}\tilde{T}_z &  = \frac{1}{{\rho c_{p} }} \left( {k(\tilde{T})\tilde{T}_z} \right)_z + \frac{{D_{T} k_{t}^{*} }}{{c_{s} c_{p} }}\tilde{C}_{zz} + \tau \left( {D_{B} \tilde{C}_z \tilde{T}_z + \frac{{D_{T} }}{{\tilde{T}_{\infty } }}\left( { \tilde{T}_z} \right)^{2} } \right) \\     & \quad  - \frac{1}{{\rho c_{p} }} {q_z}(r) + \frac{{\rho _{p} c_{p} }}{{\left( {\rho c_{p} } \right)_{f} \tau _{T} }}\left( {\tilde{T}_{P}  - \tilde{T}} \right), \\  \end{aligned} $$

7 $$ \begin{gathered}   \tilde{u}\tilde{C}_x + \tilde{v}\tilde{C}_y + \tilde{w}\tilde{C}_y = \left( {D_{B} (\tilde{C})\tilde{C}_z} \right)_z + \frac{{D_{T} k_{t}^{*} }}{{\tilde{T}_{\infty } }}\tilde{T}_{zz} + \frac{{\rho _{p} }}{{\rho \tau _{c} }}\left( {C_{p}  - C} \right) - k_{r}^{2} \left( {\frac{{\tilde{T}}}{{\tilde{T}_{\infty } }}} \right)^{n} \left( {\tilde{C} - \tilde{C}_{\infty } } \right)\exp \left( {\frac{{ - E_{a} }}{{k\tilde{T}}}} \right). \hfill \\    \hfill \\  \end{gathered} $$

The mathematical form of radiative heat flux^31,64^ is as follows:

8 $$ q_{r}  =  - \frac{4}{3}\frac{{\bar{\sigma }}}{{\bar{k}}}T^{4}_z ,\quad {\text{where }}T^{4}  = 4T_{\infty }^{3} T - 3T_{\infty }^{4} . $$

In Eq. (6), temperature-dependent thermal conductivity^16,65^ is stated as:

9 $$ k\left( T \right) = k_{\infty } \left( {1 + d\left( {\frac{{\tilde{T} - \tilde{T}_{{_{\infty } }} }}{{\tilde{T}_{w}  - \tilde{T}_{\infty } }}} \right)} \right). $$

In Eq. (7), variable molecular diffusivity^49^ is expressed as:

10 $$ D_{B} \left( C \right) = D_{{B_{\infty } }} \left( {1 + e\left( {\frac{{\tilde{C} - \tilde{C}_{\infty } }}{{\tilde{C}_{w}  - \tilde{C}_{\infty } }}} \right)} \right). $$

For dusty particle flow:

11 $$ u_{x}(p)  + v_{y}(p)  + w_{z}(p)  = 0, $$

12 $$ u(p) u_x(p) + v(p) u_y + w(p) u_z = \frac{{KN}}{{\rho p}}\left( {u - u(p) } \right), $$

13 $$ u(p)v_x(p)  + v(p) v_y(p) + w(p) v_z(p) = \frac{{KN}}{{\rho_ p }}\left( {v - v(p) } \right), $$

14 $$ u(p) T_x(p)  + v(p) T_y(p) + w(p) T_z(p) = \frac{{c_{p} }}{{c_{m} \tau _{T} }}\left( {T - T(p)} \right), $$

15 $$ u(p) C_x(p)  + v(p)C_y(p)  + w(p)  C_z(p)  = \frac{{mN}}{{\rho \tau _{c} }}\left( {C - C(p) } \right), $$

with boundary conditions^1,7,66,67^

16 $$ \begin{array}  {cc}  \left. {\tilde{u}} \right|_{{z = 0}}  = c\left( {x + y} \right) + S\left( {1 + \frac{1}{\beta }} \right)\tilde{u}_z,{\text{  }}\left. {\tilde{v}} \right|_{{z = 0}}  = b\left( {x + y} \right) + S\left( {1 + \frac{1}{\beta }} \right)\tilde{v}_z,{\text{ }}\left. {\tilde{w}} \right|_{{z = 0}}  = 0,\left. {{\text{ }} - k_{f} \left( {\tilde{T}} \right)\tilde{T}_z} \right|_{{z = 0}}  = h_{1} \left( {\tilde{T}_{w}  - \tilde{T}} \right), \\      \left. {\tilde{C}} \right|_{{z = 0}}  = \tilde{C}_{w} , \\      \left. u \right|_{{z \to \infty }}  \to 0,{\text{  }}\left. v \right|_{{z \to \infty }}  \to 0,\left. {{\text{  }}u_{p} } \right|_{{z \to \infty }}  \to 0,\left. {{\text{  }}v_{p} } \right|_{{z \to \infty }}  \to 0,{\text{  }}\left. {w_{p} } \right|_{{z \to \infty }}  \to w,\left. T \right|_{{z \to \infty }}  \to T_{\infty } ,\left. {{\text{  }}T_{p} } \right|_{{z \to \infty }}  \to T_{\infty } ,{\text{ }}\left. C \right|_{{z \to \infty }}  \to C_{\infty } {\text{,  }} \\      \left. {C_{p} } \right|_{{z \to \infty }}  \to C_{\infty } . \\  \end{array} $$

Using appropriate subsequent transformation^10^:

17 $$ \begin{aligned}    & u = \left( {x + y} \right)cf',{\text{ }}v = \left( {x + y} \right)cj',{\text{ }}w =  - \left( {f + j} \right)\sqrt {cv} ,{\text{ }}\zeta  = \left( {\frac{c}{v}} \right)^{{0.5}} z,{\text{ }}u_{p}  = \left( {x + y} \right)cF',{\text{ }}v_{p}  = \left( {x + y} \right)cJ',{\text{ }}w_{p}  =  - \left( {F + J} \right)\sqrt {cv} , \\     & T = \left( {T_{w}  - T_{\infty } } \right)\theta  + T_{\infty } ,{\text{  }}T_{p}  = \left( {T_{w}  - T_{\infty } } \right)\theta _{p}  + T_{\infty } ,{\text{ }}C = \left( {C_{w}  - C_{\infty } } \right)\phi  + C_{\infty } ,{\text{  }}C_{p}  = \left( {C_{w}  - C_{\infty } } \right)\phi _{p}  + C_{\infty } . \\  \end{aligned} $$

Equations (3) and (11) are trivially equated. Though Eqs. (4)–(7) and (12)–(15) are transmuted as:

For fluid flow:

18 $$ \left( {1 + \frac{1}{\beta }} \right)\frac{{d^{3} f}}{{d\zeta ^{3} }} - \left( {\frac{{df}}{{d\zeta }}} \right)^{2}  - \frac{{dj}}{{d\zeta }}.\frac{{df}}{{d\zeta }} + \left( {j + f} \right)\frac{{d^{2} f}}{{d\zeta ^{2} }} - \left( {Ha + \lambda _{1} } \right)\frac{{df}}{{d\zeta }} + \lambda .\delta _{v} \left( {\frac{{dF}}{{d\zeta }} - \frac{{df}}{{d\zeta }}} \right) = 0, $$

19 $$ \left( {1 + \frac{1}{\beta }} \right)\frac{{d^{3} j}}{{d\zeta ^{3} }} - \left( {\frac{{dj}}{{d\zeta }}} \right)^{2}  - \frac{{dj}}{{d\zeta }}.\frac{{df}}{{d\zeta }} + \left( {j + f} \right)\frac{{d^{2} j}}{{d\zeta ^{2} }} - \left( {Ha + \lambda _{1} } \right)\frac{{dj}}{{d\zeta }} + \lambda .\delta _{v} \left( {\frac{{dJ}}{{d\zeta }} - \frac{{dj}}{{d\zeta }}} \right) = 0, $$

20 $$ \left( {\left( {1 + d\theta } \right) + \frac{4}{3}Rd} \right)\frac{{d^{2} \theta }}{{d\zeta ^{2} }} + d\left( {\frac{{d\theta }}{{d\zeta }}} \right)^{2}  + \Pr \left( \begin{gathered}   \left( {f + j} \right)\frac{{d\theta }}{{d\zeta }} + D_{f} \frac{{d^{2} \phi }}{{d\zeta ^{2} }} + N_{b} \frac{{d\theta }}{{d\zeta }}\frac{{d\phi }}{{d\zeta }} \hfill \\    + N_{t} \left( {\frac{{d\theta }}{{d\zeta }}} \right)^{2}  + \lambda \delta _{T} \left( {\theta _{p}  - \theta } \right) \hfill \\  \end{gathered}  \right) = 0, $$

21 $$ \left( {1 + e\phi } \right)\frac{{d^{2} \phi }}{{d\zeta ^{2} }} + e\left( {\frac{{d\phi }}{{d\zeta }}} \right)^{2}  + S_{c} \left( \begin{gathered}   \left( {f + j} \right)\frac{{d\phi }}{{d\zeta }} + \lambda \delta _{c} \left( {\phi _{p}  - \phi } \right) + S_{r} \frac{{d^{2} \theta }}{{d\zeta ^{2} }} \hfill \\    - \delta \phi \left( {1 + \alpha \theta } \right)^{n} \exp \left( {\frac{{ - E}}{{1 + \alpha \theta }}} \right) \hfill \\  \end{gathered}  \right) = 0. $$

For the dusty flow:

22 $$ \left( {J + F} \right)\frac{{d^{2} F}}{{d\zeta ^{2} }} - \left( {\frac{{dF}}{{d\zeta }}} \right)^{2}  - \frac{{dJ}}{{d\zeta }}.\frac{{dF}}{{d\zeta }} + \delta _{v} \left( {\frac{{df}}{{d\zeta }} - \frac{{dF}}{{d\zeta }}} \right) = 0, $$

23 $$ \left( {J + F} \right)\frac{{d^{2} J}}{{d\zeta ^{2} }} - \left( {\frac{{dJ}}{{d\zeta }}} \right)^{2}  - \frac{{dJ}}{{d\zeta }}.\frac{{dF}}{{d\zeta }} + \delta _{v} \left( {\frac{{dj}}{{d\zeta }} - \frac{{dJ}}{{d\zeta }}} \right) = 0, $$

24 $$ \left( {J + F} \right)\frac{{d\theta _{p} }}{{d\zeta }} + \gamma \delta _{T} \left( {\theta  - \theta _{p} } \right) = 0, $$

25 $$ \left( {F + J} \right)\frac{{d\phi _{p} }}{{d\zeta }} + \lambda .\delta _{c} \left( {\phi  - \phi _{p} } \right) = 0, $$

and the boundary conditions take the form:

$$ \begin{aligned}    & f\left( \zeta  \right) = 0,{\text{ }}j\left( \zeta  \right) = 0,{\text{ }}\frac{{df}}{{d\zeta }} = 1 + L\left( {1 + \frac{1}{\beta }} \right)\frac{{d^{2} f}}{{d\zeta ^{2} }},{\text{ }}\frac{{dj}}{{d\zeta }} = P + L\left( {1 + \frac{1}{\beta }} \right)\frac{{d^{2} j}}{{d\zeta ^{2} }},{\text{ }} \\     & \frac{{d\theta }}{{d\zeta }} =  - H_{1} \left( {\frac{{1 - \theta \left( 0 \right)}}{{1 + d\theta }}} \right),{\text{ }}\phi \left( \zeta  \right) = 1{\text{ }}\quad {\text{at }}\,{\text{  }}\zeta {\text{ = 0,}} \\  \end{aligned} $$

26 $$ \begin{aligned}    & \frac{{df}}{{d\zeta }} \to 0,\frac{{dj}}{{d\zeta }} \to 0,\frac{{dF}}{{d\zeta }} \to 0,\frac{{dJ}}{{d\zeta }} \to 0,F\left( \zeta  \right) \to f\left( \zeta  \right), \\     & J\left( \zeta  \right) \to j\left( \zeta  \right),\theta \left( \zeta  \right) \to 0,\theta _{p} \left( \zeta  \right) \to 0,\phi \left( \zeta  \right) \to 0,{\text{ }}\phi _{p} \left( \zeta  \right) \to 0{\text{ }}\quad {\text{  as  }}\,\zeta  \to \infty . \\  \end{aligned} $$

The mathematical forms of shear stress at the wall (drag force coefficient), local Nusselt, and Sherwood number are specified as:

27 $$ C_{{f_{x} }}  = \frac{{\left. {\tau ({{zx}}) } \right|_{{z = 0}} }}{{\rho u_{w}^{2} }},\,\tau ({{zx}})  = \mu \left( {1 + \frac{1}{\beta }} \right)u_z, $$

28 $$ C_{{j_{y} }}  = \frac{{\left. {\tau ({{zy}}) } \right|_{{z = 0}} }}{{\rho u_{w}^{2} }},\,\tau ({{zy}})  = \mu \left( {1 + \frac{1}{\beta }} \right)v_z, $$

29 $$ Nu({x})  = \frac{{xQ_{w} }}{{k_{\infty } \left( {\tilde{T}_{w}  - \tilde{T}_{\infty } } \right)}},\,Q_{w}  = \left. { - k\left( T \right)T_z + q(r) } \right|_{{z = 0}} , $$

30 $$ Sh({x})  = \frac{{xQ_{m} }}{{D_{{B_{\infty } }} \left( {C_{w}  - C_{\infty } } \right)}},\,Q_{m}  =  - D_{B} \left( C \right)\left. { C_{z} } \right|_{{z = 0}} . $$

By employing Eq. (17), the dimensionless form of Eqs. (27)–(30) are as follow:

31 $$ \left( {\text{Re} _{x} } \right)^{{0.5}} C_{{f_{x} }}  = \left( {1 + \frac{1}{\beta }} \right)\left. {\frac{{d^{2} f}}{{d\zeta ^{2} }}} \right|_{{\zeta  = 0}} , $$

32 $$ \left( {\text{Re} _{x} } \right)^{{0.5}} C_{{j_{y} }}  = \left( {1 + \frac{1}{\beta }} \right)\left. {\frac{{d^{2} j}}{{d\zeta ^{2} }}} \right|_{{\zeta  = 0}} , $$

33 $$ Nu({x}) \left( {\text{Re} _{x} } \right)^{{ - 0.5}}  =  - \left( {1 + \frac{4}{3}\left( {\frac{{Rd}}{{1 + d\theta }}} \right)} \right)\left. {\frac{{d\theta }}{{d\zeta }}} \right|_{{\zeta  = 0}} , $$

34 $$ Sh({x}) \left( {\text{Re} _{x} } \right)^{{ - 0.5}}  =  - \frac{1}{{\left( {1 + e\phi \left( \zeta  \right)} \right)}}\left. {\frac{{d\phi }}{{d\zeta }}} \right|_{{\zeta  = 0}} . $$

## Numerical procedure

The coupled nonlinear ODEs are computed numerically by employing the bvp4c function in MATLAB. Mentioned numerical code is used, we obtain ODEs which are of order one.

$$ \begin{aligned}    & f = Y_{1} ,f' = Y_{2} ,f'' = Y_{3} ,f''' = Y_{3} ^{\prime }  = YY_{1} ,F = Y_{4} ,F' = Y_{5} ,F'' = Y_{5} ' = YY_{2} , \\     & j = Y_{6} ,j' = Y_{7} ,j'' = Y_{8} ,j' = Y_{8} ^{\prime }  = YY_{3} ,J = Y_{9} ,J' = Y_{{10}} ,J'' = Y_{{10}} ^{\prime }  = YY_{4} , \\     & YY_{1}  = \frac{1}{{\left( {1 + \frac{1}{\beta }} \right)}}\left( {Y_{2}^{2}  + Y_{7}  \cdot Y_{2}  - \left( {Y_{1}  + Y_{6} } \right)Y_{3}  + \left( {Ha + \lambda _{1} } \right)Y_{2}  - \lambda  \cdot \delta _{v} \left( {Y_{5}  - Y_{2} } \right)} \right), \\     & YY_{2}  = \frac{1}{{\left( {Y_{9}  + Y_{4} } \right)}}\left( {Y_{5}^{2}  + Y_{{10}}  \cdot Y_{5}  - \delta _{v} \left( {Y_{2}  - Y_{5} } \right)} \right), \\     & YY_{3}  = \frac{1}{{\left( {1 + \frac{1}{\beta }} \right)}}\left( {Y_{7}^{2}  + Y_{7}  \cdot Y_{2}  - \left( {Y_{6}  + Y_{1} } \right)Y_{8}  + \left( {Ha + \lambda _{1} } \right)Y_{7}  - \lambda  \cdot \delta _{v} \left( {Y_{{10}}  - Y_{7} } \right)} \right), \\     & YY_{4}  = \frac{1}{{\left( {Y_{9}  + Y_{4} } \right)}}\left( {Y_{{10}}^{2}  + Y_{{10}}  \cdot Y_{5}  - \delta _{v} \left( {Y_{7}  - Y_{{10}} } \right)} \right), \\     & \theta  = Y_{{11}} ,\theta ' = Y_{{12}} ,\theta '' = Y_{{12}} ^{\prime }  = YY_{5} ,\theta _{p}  = Y_{{13}} ,\theta _{p} ^{\prime }  = Y_{{13}} ^{\prime }  = YY_{6} , \\     & \phi  = Y_{{14}} ,\phi  = Y_{{15}} ,\phi '' = Y_{{15}} ^{\prime }  = YY_{7} , \\     & \phi _{p}  = Y_{{16}} ,\phi _{p} ^{\prime }  = Y_{{16}} ^{\prime }  = YY_{8} . \\     & YY_{8}  = \frac{1}{{\left( {Y_{9}  + Y_{4} } \right)}}\left( {\lambda  \cdot \delta _{c} \left( {Y_{{16}}  - Y_{{14}} } \right)} \right) \\     & YY_{5}  = \frac{1}{{\left( {\left( {1 + d \cdot Y_{{11}} } \right) + \frac{4}{3}Rd} \right)}}\left( { - d \cdot Y_{{12}}^{2}  - \Pr \left( \begin{gathered}   \left( {Y_{1}  + Y_{6} } \right)Y_{{12}}  + D_{f} .YY_{7}  + N_{b}  \cdot Y_{{12}}  \cdot Y_{{15}}  \hfill \\    + N_{t}  \cdot Y_{{12}}^{2}  + \lambda  \cdot \delta _{T} \left( {Y_{{13}}  - Y_{{11}} } \right) \hfill \\  \end{gathered}  \right)} \right), \\     & YY_{6}  = \frac{1}{{\left( {Y_{9}  + Y_{4} } \right)}}\left( { - \gamma  \cdot \delta _{T} \left( {Y_{{11}}  - Y_{{13}} } \right)} \right), \\     & YY_{7}  = \frac{1}{{\left( {1 + e \cdot Y_{{14}} } \right)}}\left( { - e \cdot Y_{{15}}^{2}  + S_{c} \left( \begin{gathered}   \delta  \cdot Y_{{14}} \left( {1 + \alpha  \cdot Y_{{11}} } \right)^{n} \exp \left( {\frac{{ - E}}{{1 + \alpha  \cdot Y_{{11}} }}} \right) \hfill \\    - \left( {Y_{1}  + Y_{6} } \right)Y_{{15}}  - S_{r}  \cdot YY_{5}  - \lambda  \cdot \delta _{c} \left( {Y_{{16}}  - Y_{{14}} } \right) \hfill \\  \end{gathered}  \right)} \right), \\  \end{aligned} $$

35 $$ \begin{aligned}    & {\text{and }}\,{\text{the}}\,{\text{ boundary }}\,{\text{conditions }}\,{\text{are}}\,{\text{ enumerated}}\,{\text{ as}} \\     & Y_{1} (0) = 0,Y_{6} (0) = 0,Y_{2} (0) = 1 + L \cdot \left( {1 + \frac{1}{\beta }} \right)Y_{3} (0),Y_{7} (0) = P + L \cdot \left( {1 + \frac{1}{\beta }} \right)Y_{8} (0), \\     & Y_{{12}} (0) =  - H_{1} \left( {\frac{{\left( {1 - Y_{{11}} \left( 0 \right)} \right)}}{{1 + d \cdot Y_{{11}} \left( 0 \right)}}} \right),Y_{{14}} (0) = 1\quad {\text{ At }}\,\zeta {\text{ = 0}} \\     & Y_{2} (\infty ) \to 0,Y_{7} (\infty ) \to 0,Y_{5} (\infty ) \to 0,Y_{{10}} (\infty ) \to 0,Y_{4} (\infty ) \to Y_{1} (\infty ), \\     & Y_{9} (\infty ) \to Y_{6} (\infty ),Y_{{11}} (\infty ) \to 0,Y_{{13}} (\infty ) \to 0,Y_{{14}} (\infty ) \to 0,Y_{{16}} (\infty ) \to 0.\quad {\text{As }}\,{\text{ }}\zeta  \to \infty . \\  \end{aligned} $$

## Analysis of results

**Figure 2:**
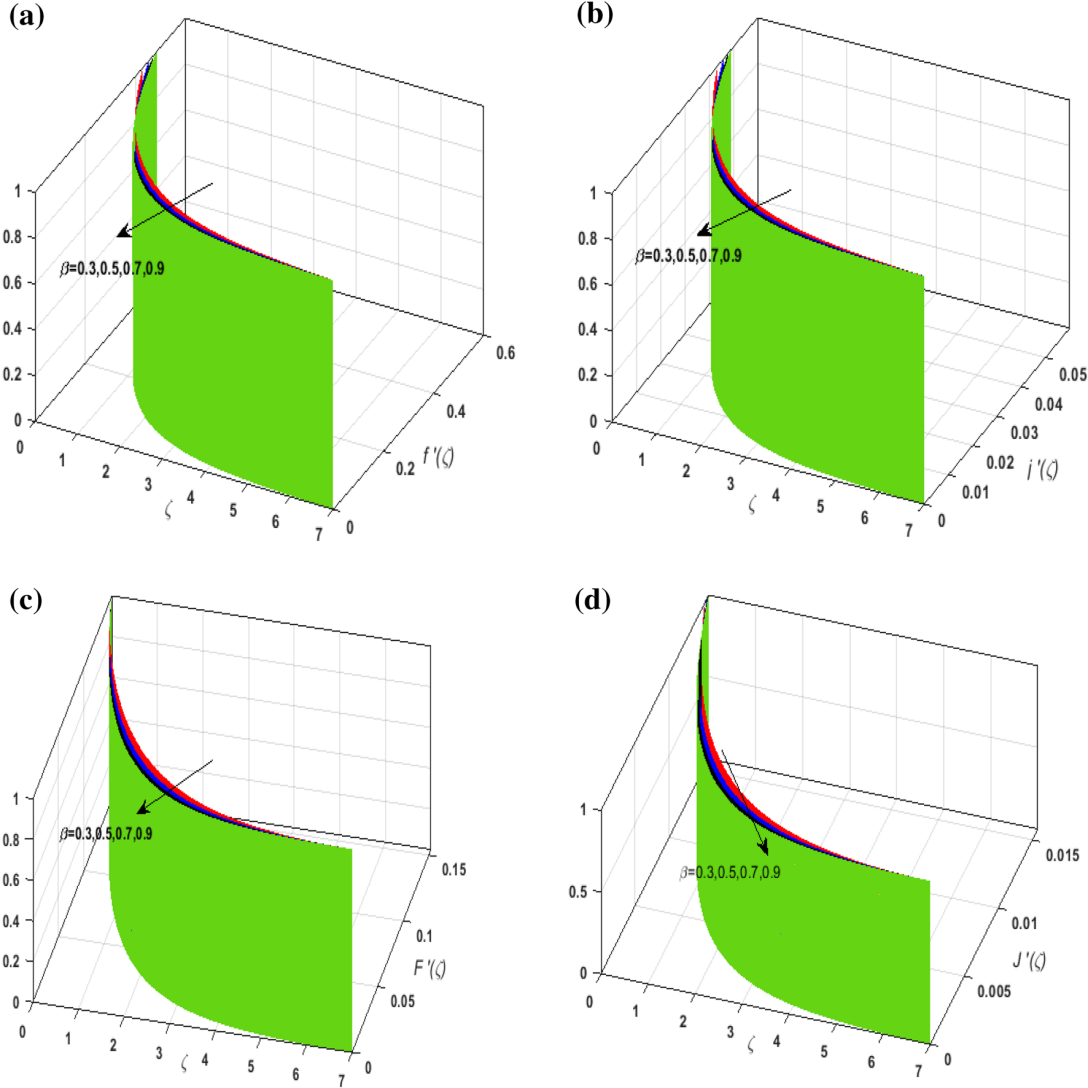
(**a**) Upshot of $\beta$ on $f'\left( \zeta  \right).$ (**b**) Upshot of $\beta$ on $j'\left( \zeta  \right).$ (**c**) Upshot of $\beta$ on $F'\left( \zeta  \right).$ (**d**) Upshot of $\beta$ on $J'\left( \zeta  \right).$

**Figure 3:**
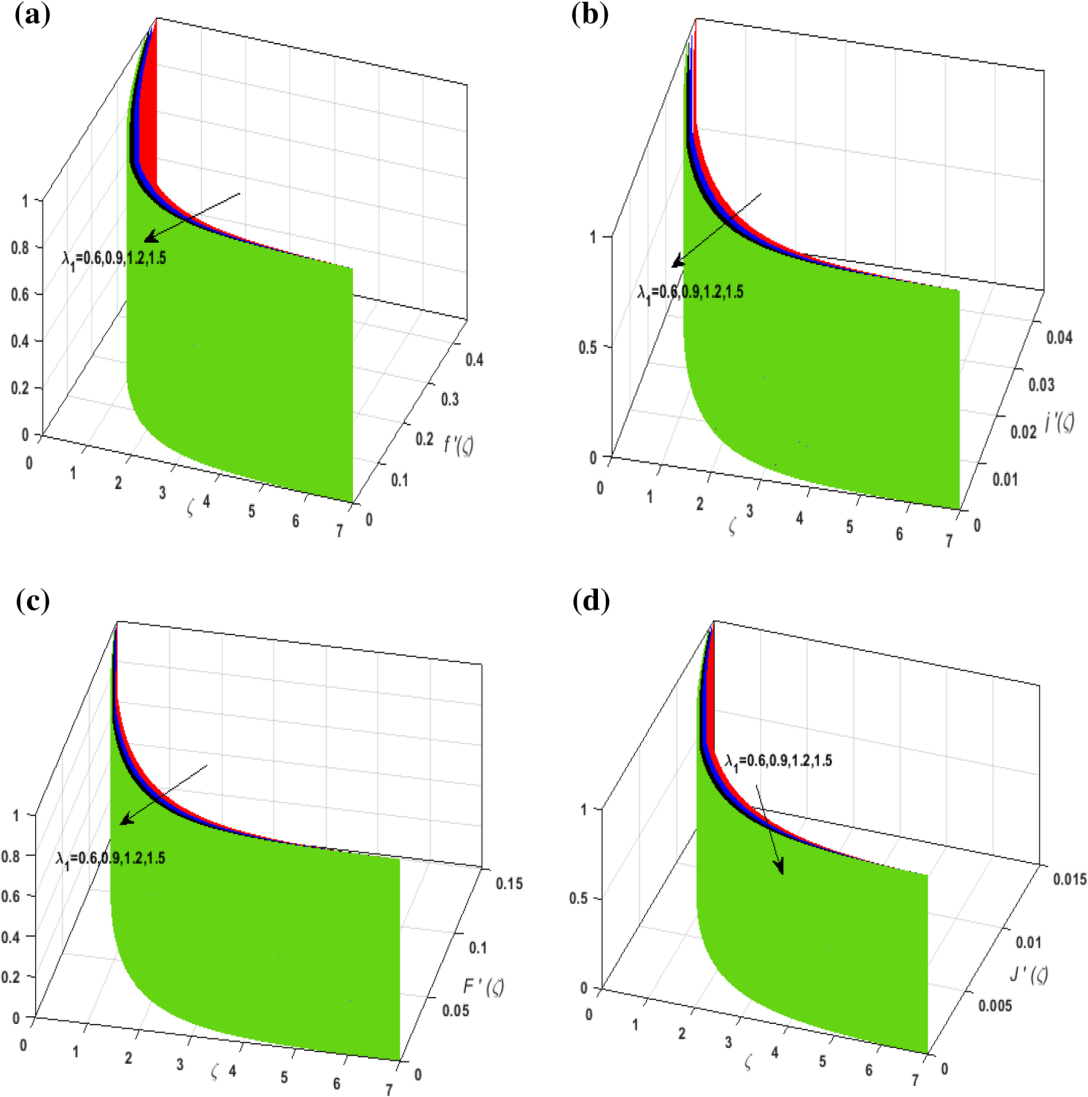
(**a**) Upshot of $\lambda _{1}$ on $f'\left( \zeta  \right).$ (**b**) Upshot of $\lambda _{1}$ on $j'\left( \zeta  \right).$ (**c**) Upshot of $\lambda _{1}$ on $F'\left( \zeta  \right).$ (**d**) Upshot of $\lambda _{1}$ on $J'\left( \zeta  \right).$

**Figure 4:**
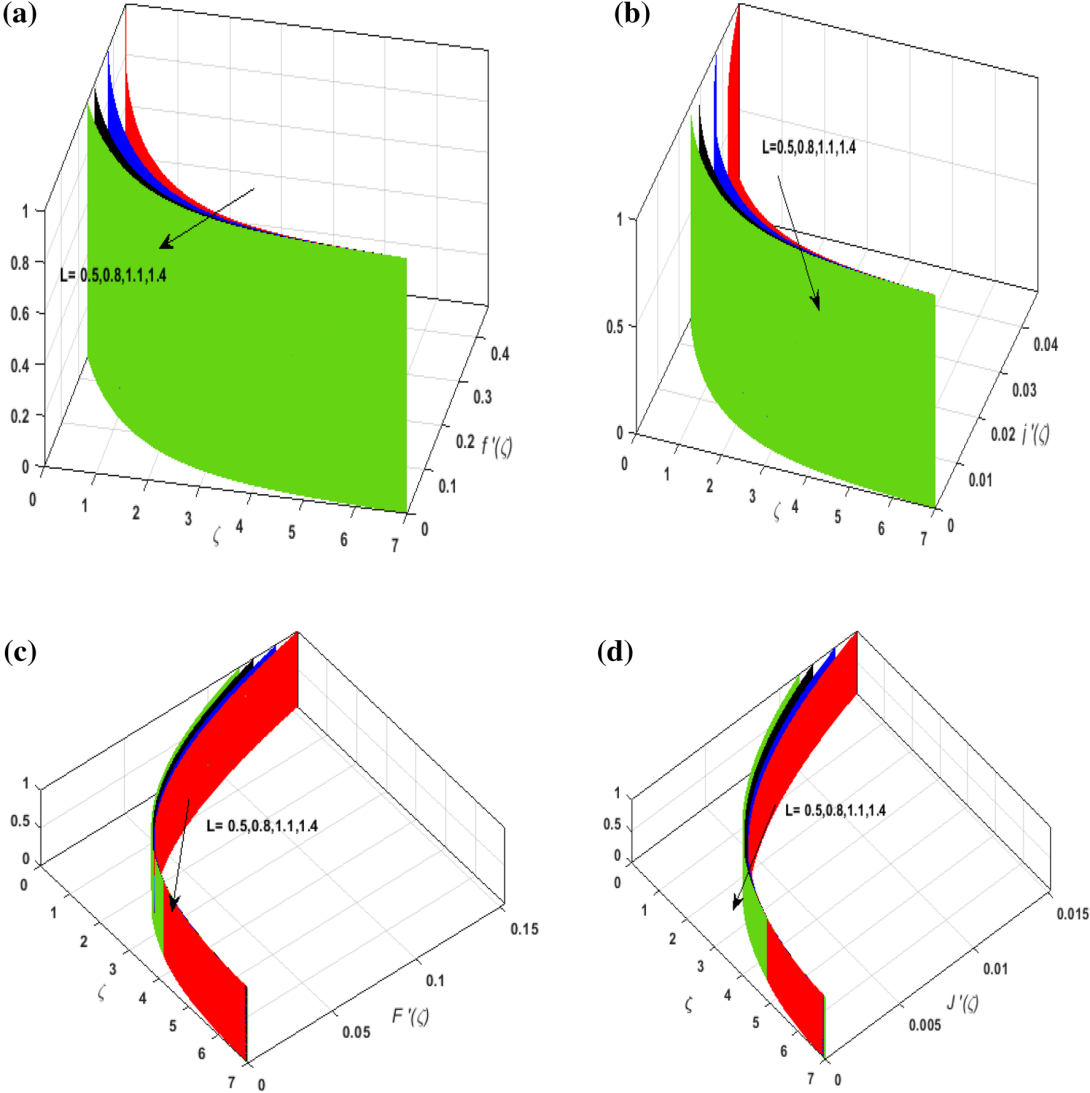
(**a**) Upshot of $L$ on $f'\left( \zeta  \right).$ (**b**) Upshot of $L$ on $j'\left( \zeta  \right).$ (**c**) Upshot of $L$ on $F'\left( \zeta  \right).$ (**d**) Upshot of $L$ on $J'\left( \zeta  \right).$

**Figure 5:**
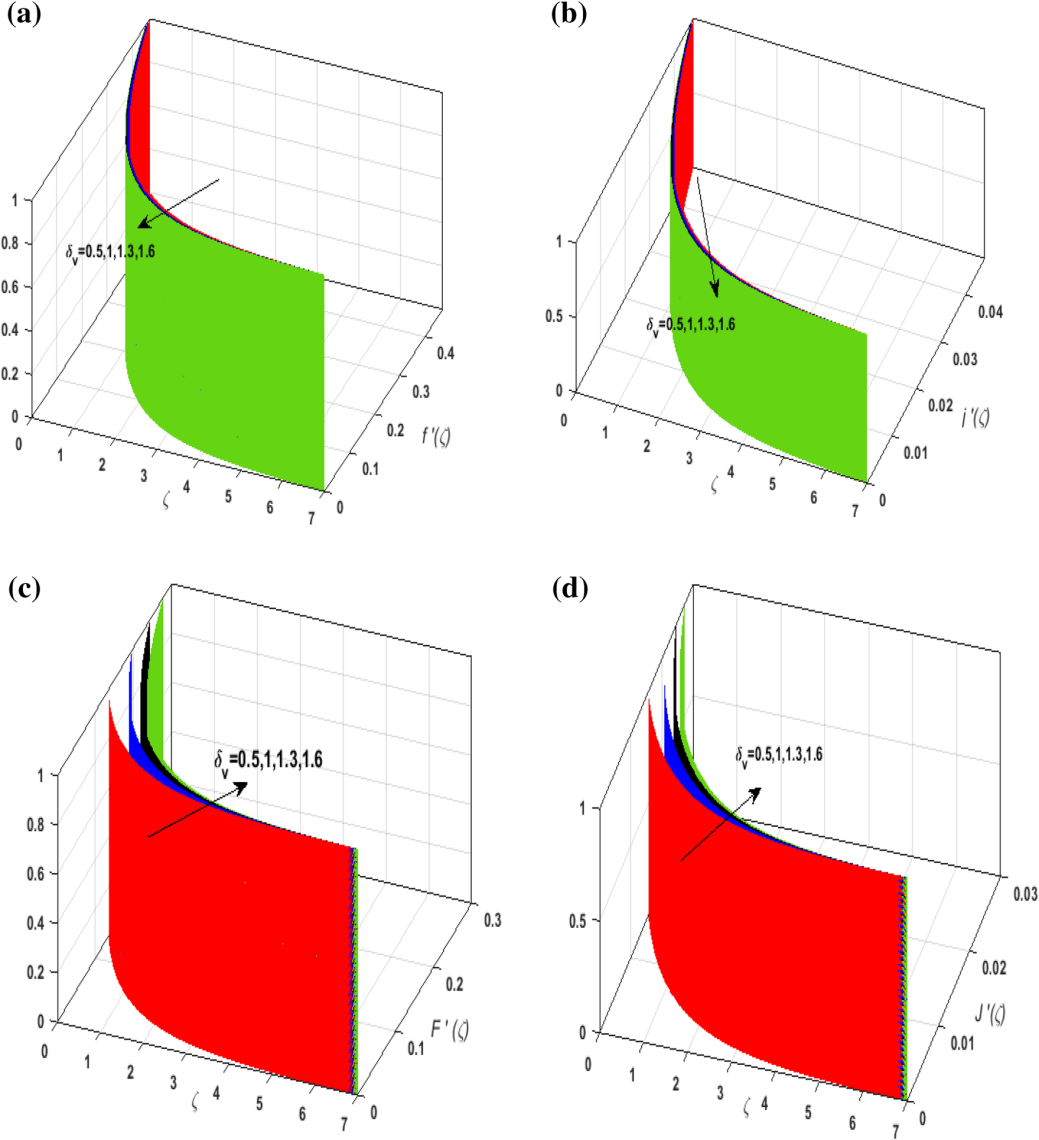
(**a**) Upshot of $L$ on $f'\left( \zeta  \right).$ (**b**) Upshot of $L$ on $j'\left( \zeta  \right).$ (**c**) Upshot of $L$ on $F'\left( \zeta  \right).$ (**d**) Upshot of $L$ on $J'\left( \zeta  \right).$

**Figure 6:**
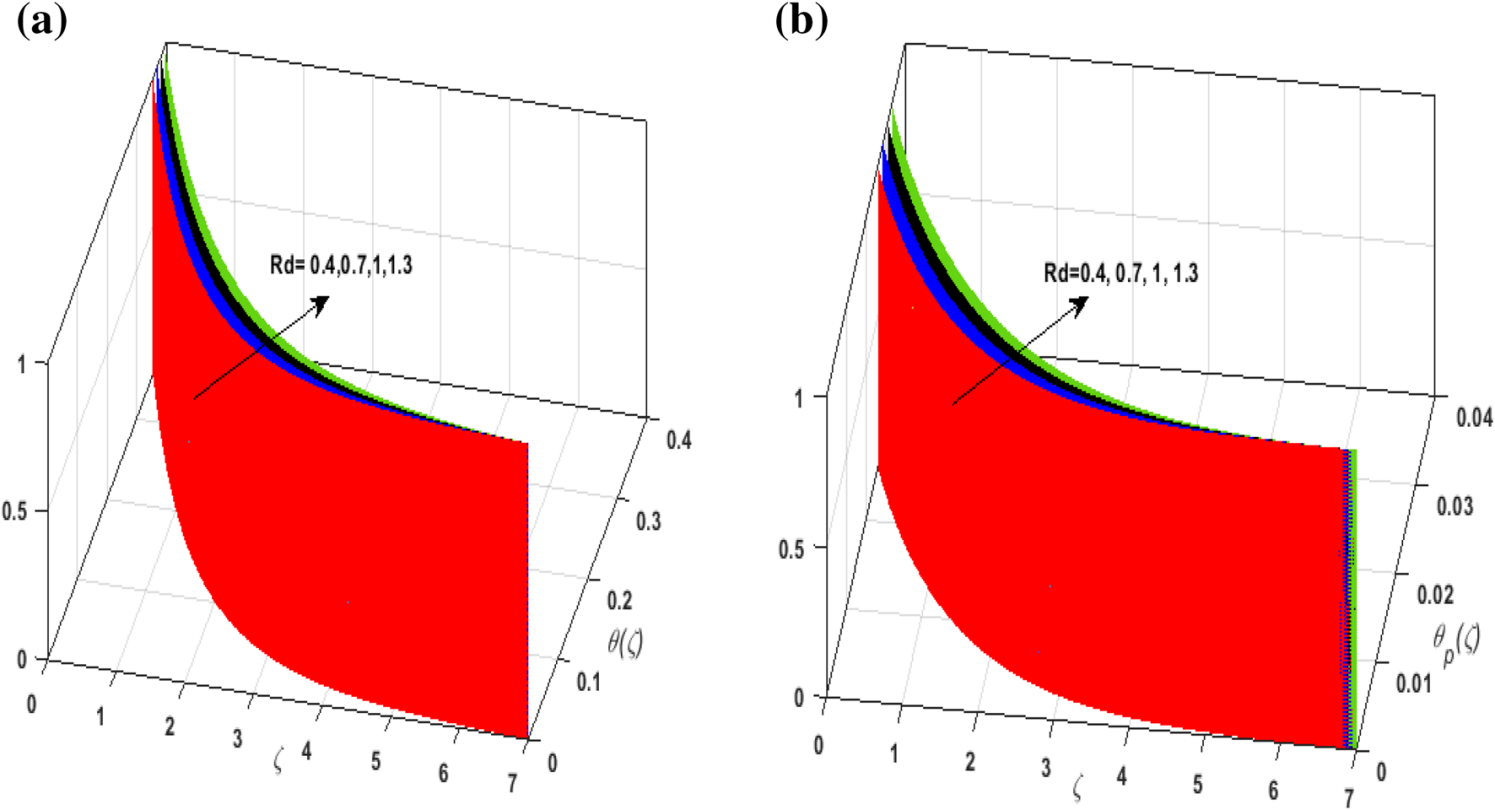
(**a**) Upshot of $Rd$ on $\theta \left( \zeta  \right).$ (**b**) Upshot of $Rd$ on $\theta _{p} \left( \zeta  \right).$

**Figure 7:**
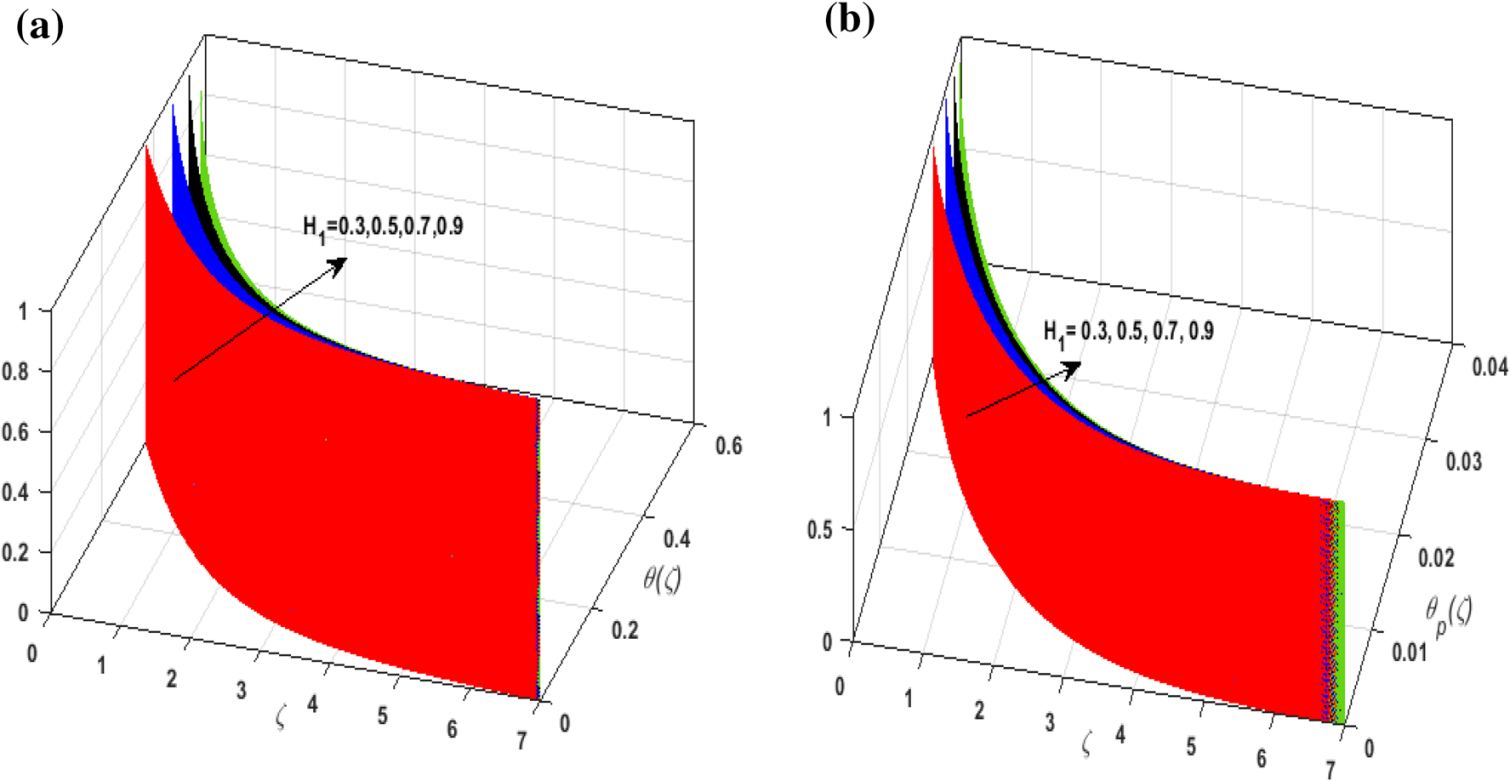
(**a**) Upshot of $H_{1}$ on $\theta \left( \zeta  \right).$ (**b**) Upshot of $H_{1}$ on $\theta _{p} \left( \zeta  \right).$

**Figure 8:**
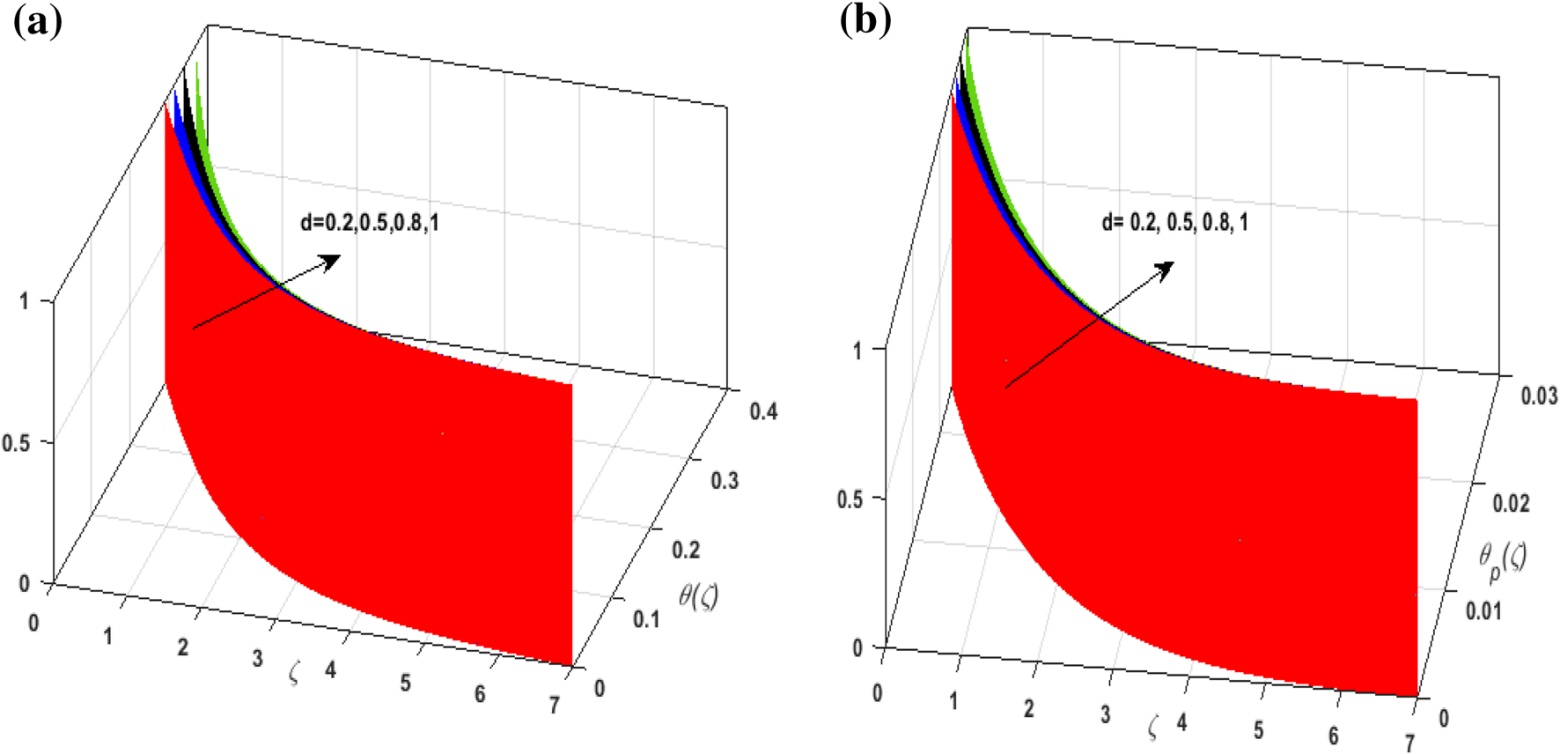
(**a**) Upshot of $d$ on $\theta \left( \zeta  \right).$ (**b**) Upshot of $d$ on $\theta _{p} \left( \zeta  \right).$

**Figure 9:**
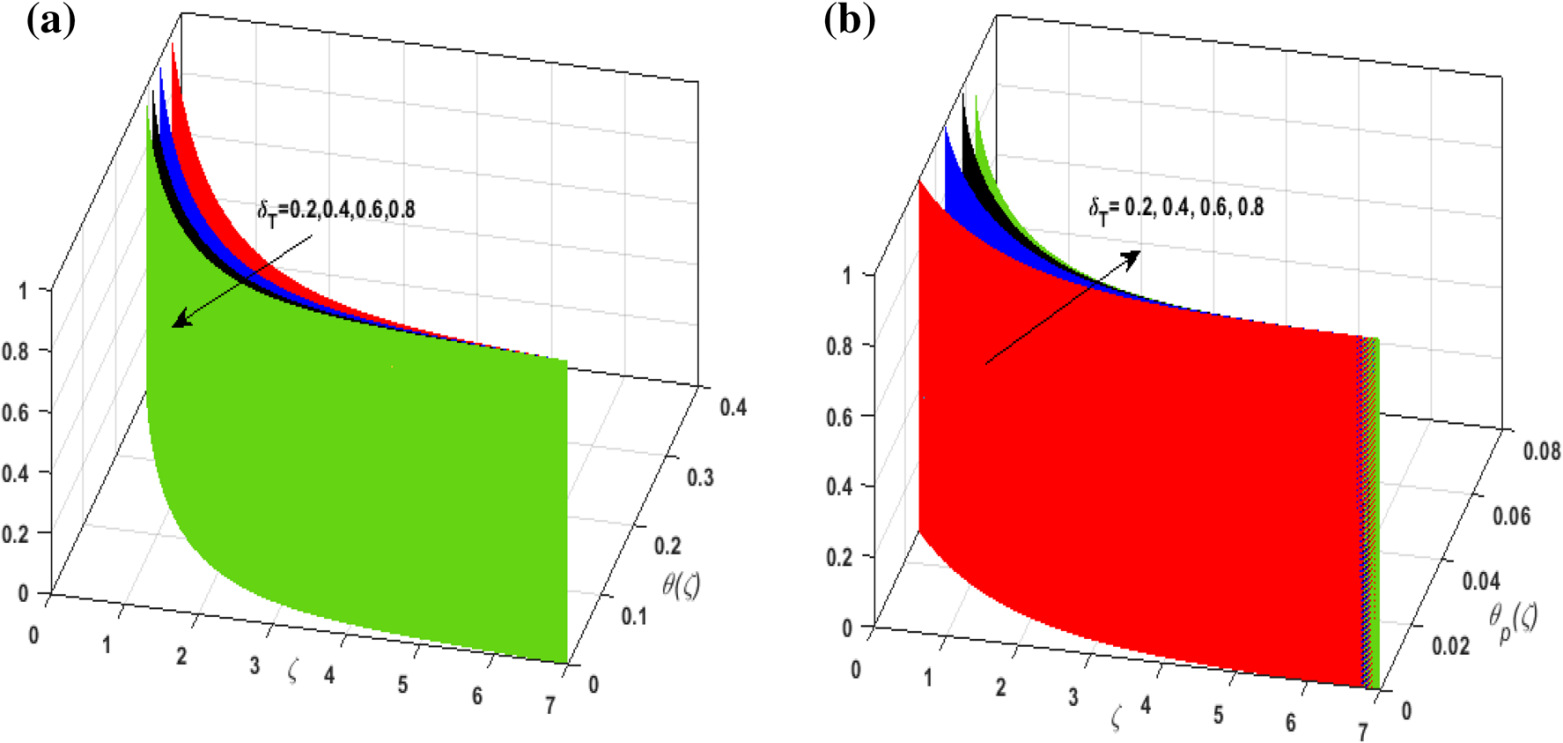
(**a**) Upshot of $\delta _{T}$ on $\theta \left( \zeta  \right).$ (**b**) Upshot of $\delta _{T}$ on $\theta _{p} \left( \zeta  \right).$

**Figure 10:**
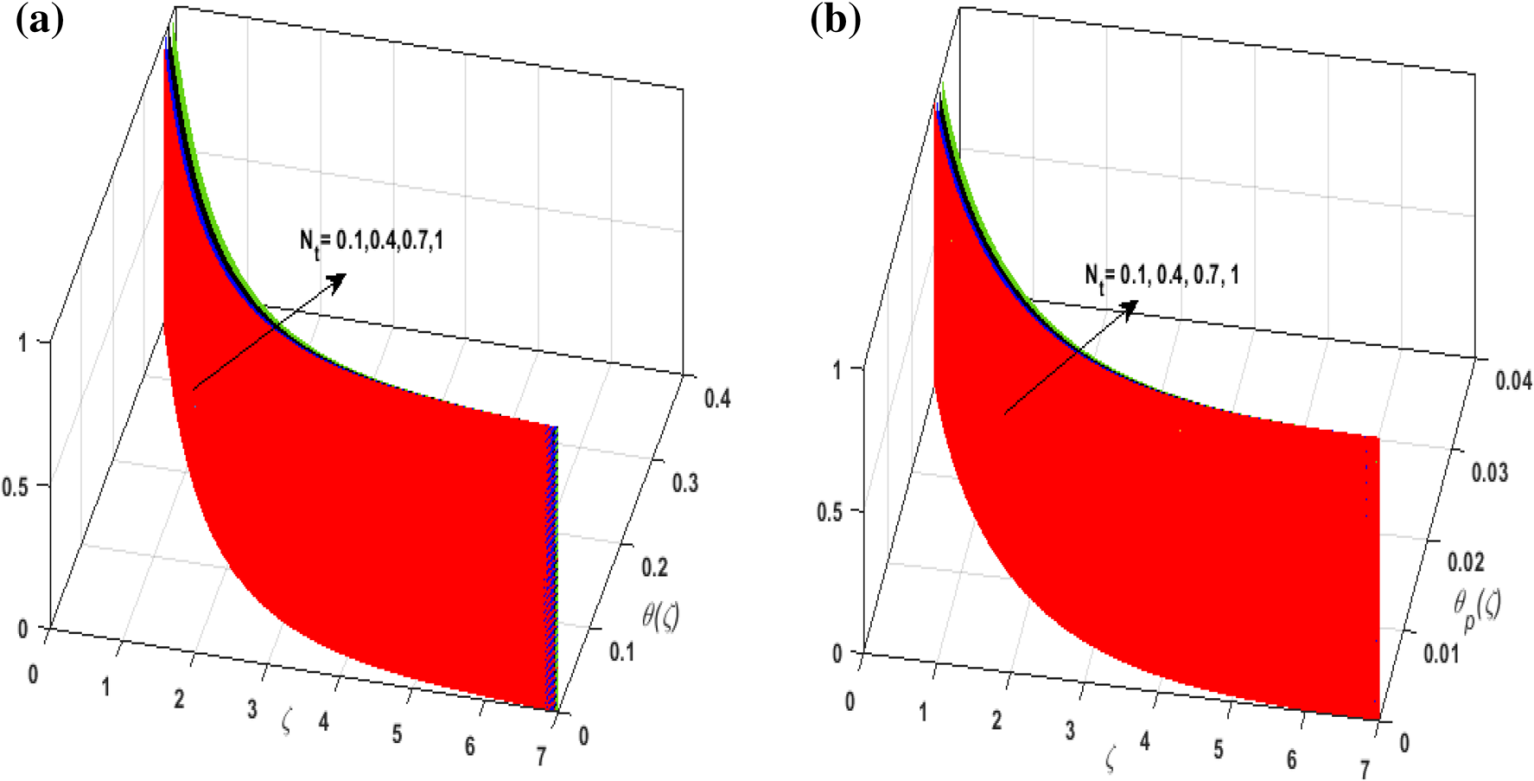
(**a**) Upshot of $N_{t}$ on $\theta \left( \zeta  \right).$ (**b**) Upshot of $N_{t}$ on $\theta _{p} \left( \zeta  \right).$

**Figure 11:**
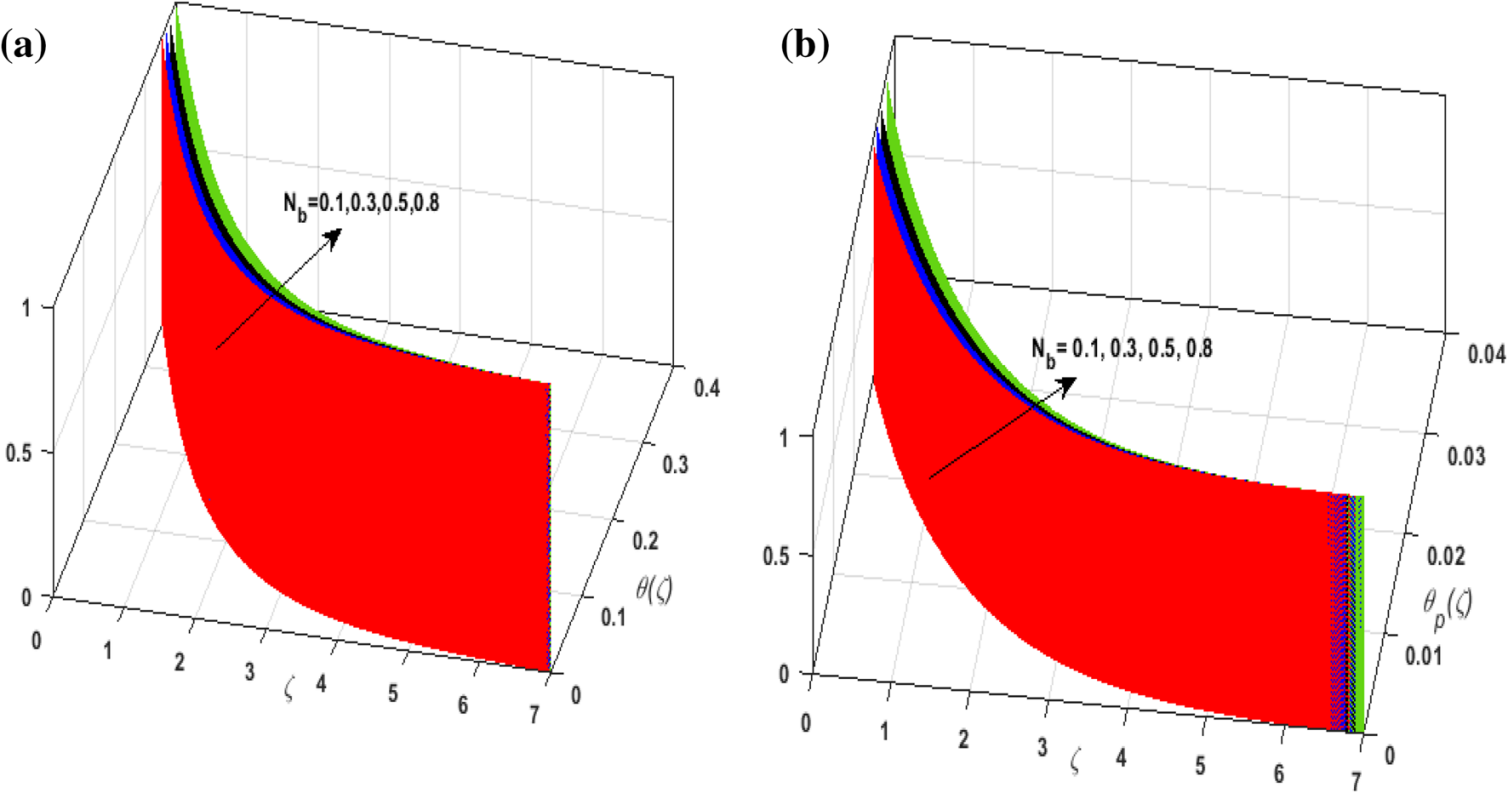
(**a**) Upshot of $N_{b}$ on $\theta \left( \zeta  \right).$ (**b**) Upshot of $N_{b}$ on $\theta _{p} \left( \zeta  \right).$

**Figure 12:**
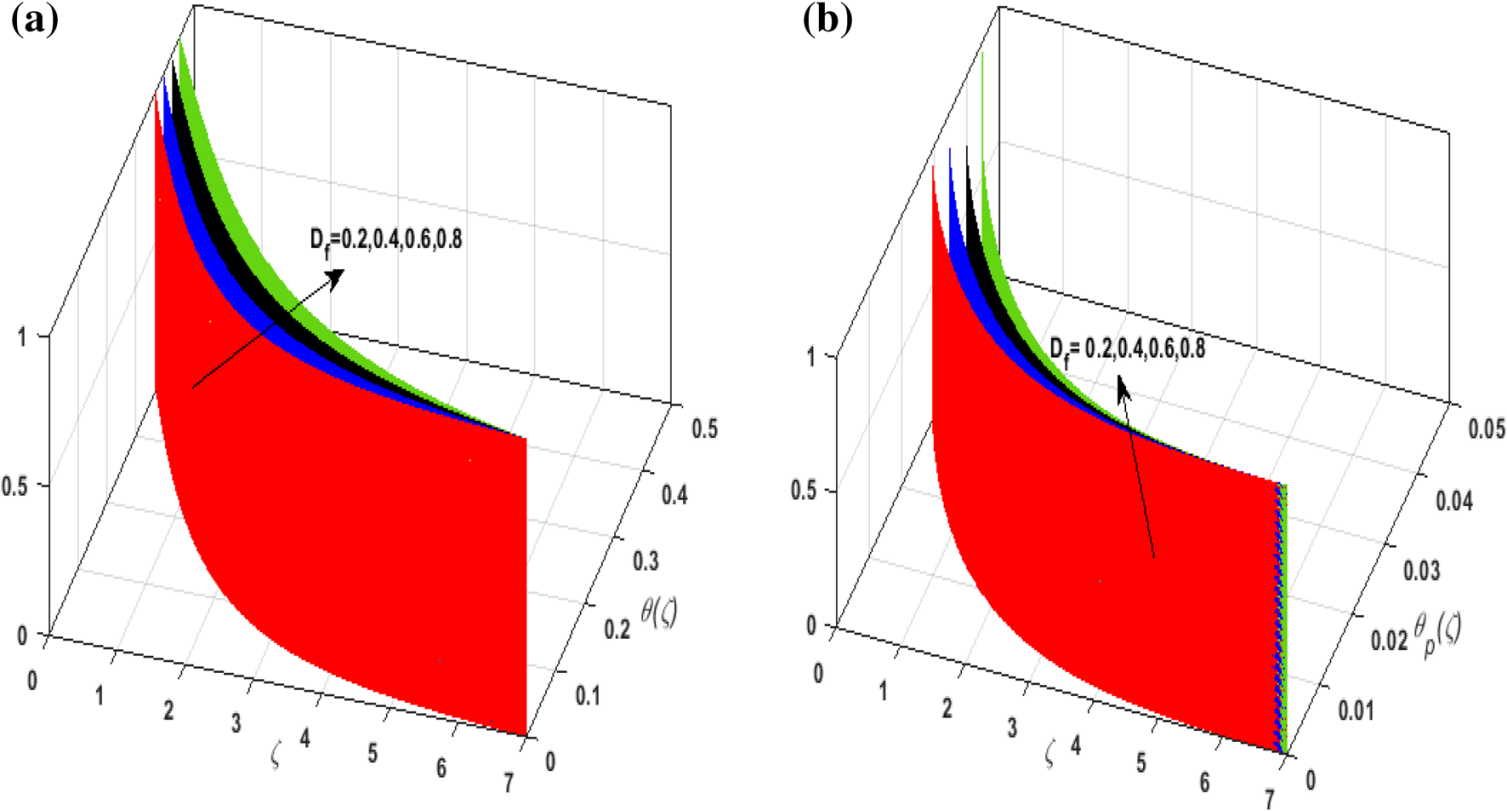
(**a**) Upshot of $D_{f}$ on $\theta \left( \zeta  \right).$ (**b**) Upshot of $D_{f}$ on $\theta _{p} \left( \zeta  \right).$

**Figure 13:**
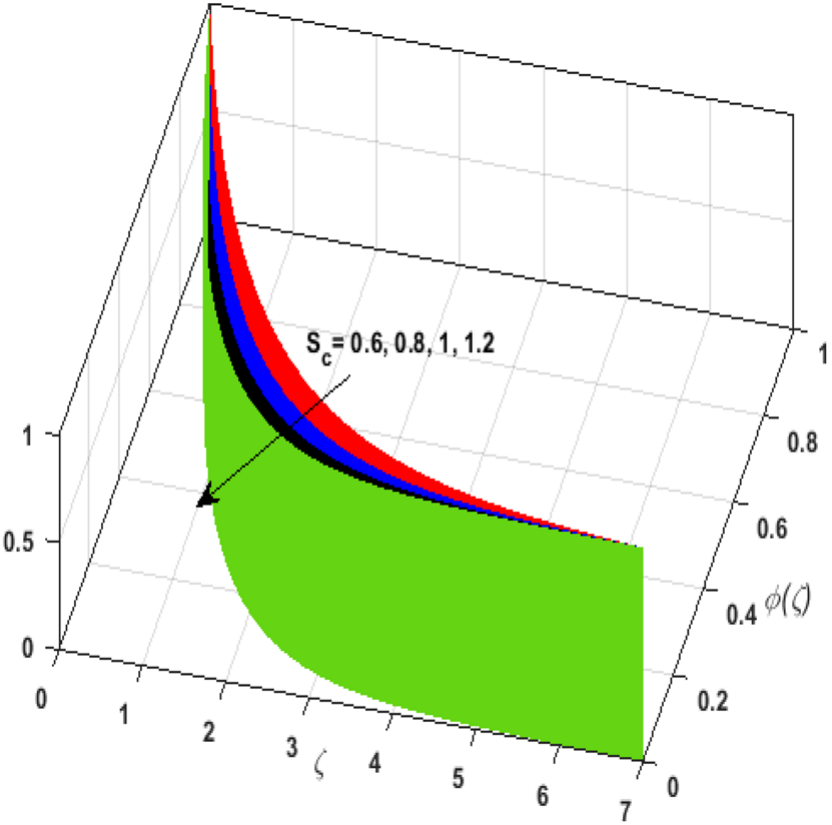
Upshot of $S_{c}$ on $\phi \left( \zeta  \right).$

**Figure 14:**
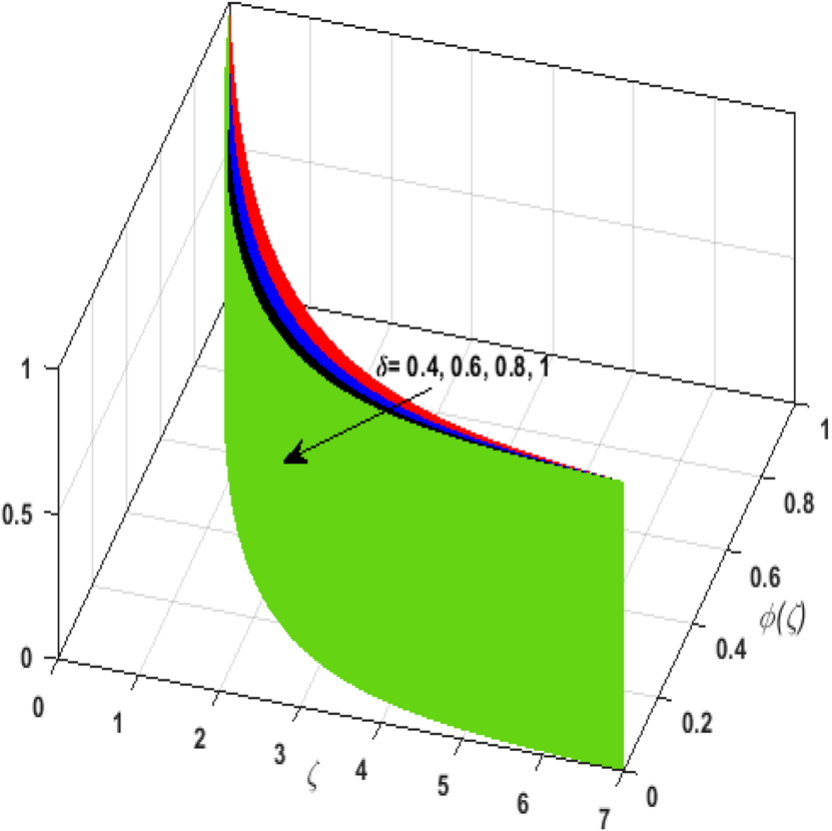
Upshot of $\delta$ on $\phi \left( \zeta  \right).$

**Figure 15:**
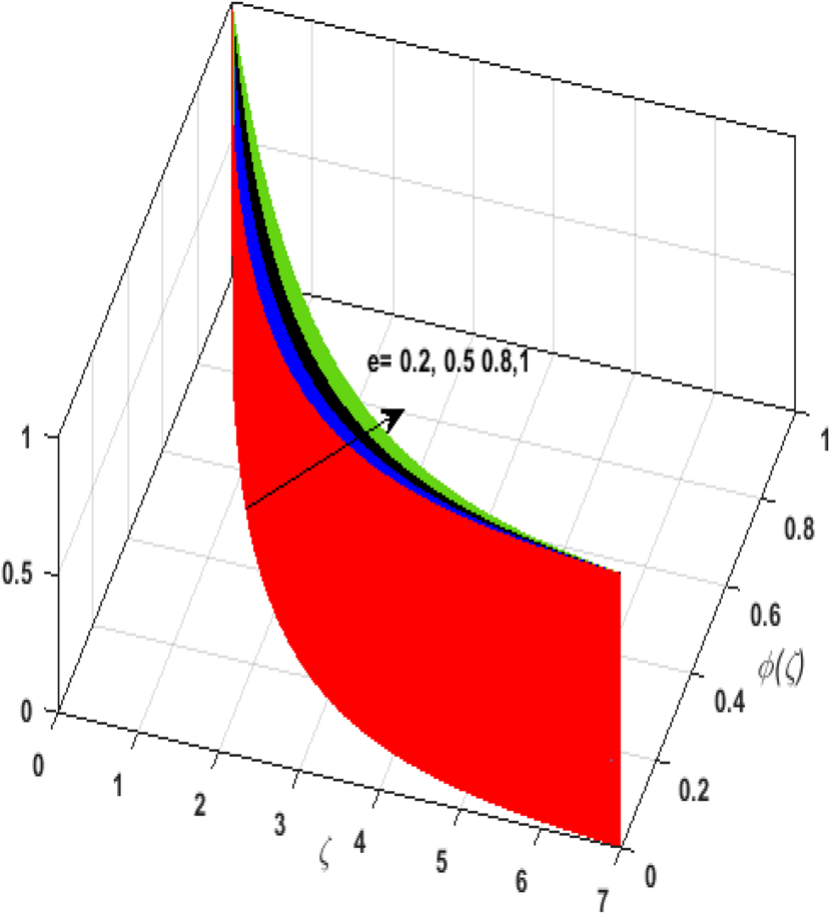
Upshot of $e$ on $\phi \left( \zeta  \right).$

**Figure 16:**
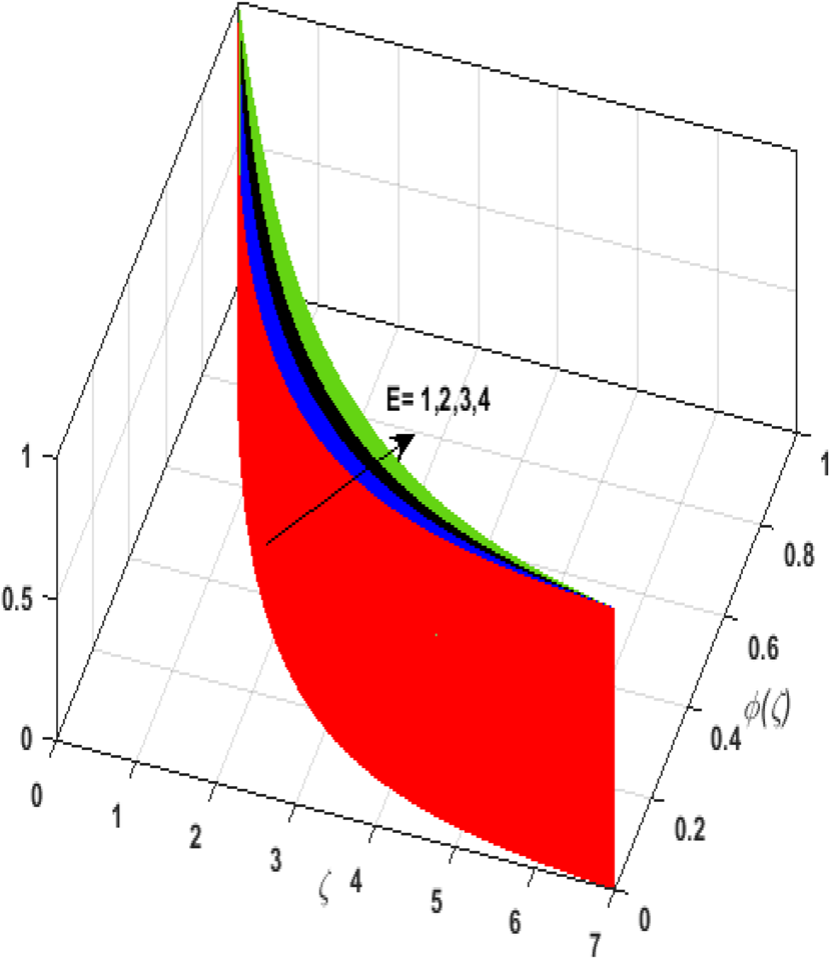
Upshot of $E$ on $\phi \left( \zeta  \right).$

**Figure 17:**
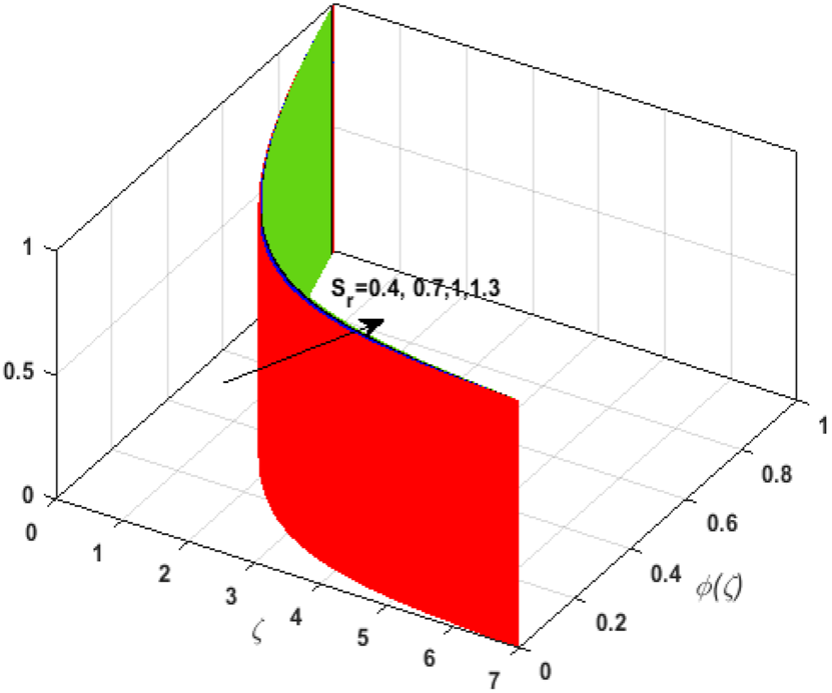
Upshot of $S_{r}$ on $\phi \left( \zeta  \right).$

For the graphical analysis of the dimensionless parameters versus involved profiles appearing in the highly nonlinear mathematical problem in Eqs. (18)–(25). This problem is elucidated numerically by utilizing bvp4c, an implemented function in MATLAB. Figures 2, 3, 4, 5 demonstrate the influence of Casson fluid parameter $$\beta$$, porosity parameter $$\lambda _{1}$$, velocity slip parameter $$L$$, and fluid-particle interaction parameter $$\delta _{v}$$ on the velocity of the fluid $$f^{\prime}\left( \zeta  \right),j^{\prime}\left( \zeta  \right)$$ (in $$x$$ and $$y$$ direction) and dust phase $$F'\left( \zeta  \right){\text{ }}and{\text{ }}J'\left( \zeta  \right).$$ The aftermath of $$\beta$$ on velocity field for both phases is illustrated in Fig. 2a–d. These figures depict that $$\beta$$ is inversely proportional to yield stress $$S_{y}$$. It is found that on escalating $$\beta$$ yield stress decreases. This generates a resistive force that causes hindrance to the fluid flow. Consequently, both phases deteriorate as $$\beta$$ escalate. The effect of the porosity parameter $$\lambda _{1}$$ on fluid and dust phase is illustrated in Fig. 3a–d. Since $$\lambda _{1}$$ is the quotient of kinematic viscosity to the permeability of the porous medium. Growing values of $$\lambda _{1}$$ escalates the kinematic viscosity of the fluid. This accelerates the resistance in the system. It is witnessed that rising values of $$\lambda _{1}$$ results in deterrence to the motion of the fluid. Therefore, the velocity field for both phases diminishes. Figure 4a–d are sketched to depict the impact of slip parameters $$L$$ on both phases. It is found that growing values of $$H_{1}$$ strengthens the friction force. This causes more liquid to slip past the deformable bidirectional surface. Thus, the fluid flow depreciates for both phases. The impression of $$\delta _{v}$$ on both phases is illustrated in Fig. 5a–d. It is observed that for rising values of $$\delta _{v}$$ relaxation time of suspended particles decays. Dusty granules generate a force that will resist the flow. Therefore, fluid velocity depreciates on mounting $$\delta _{v}$$, however, an opposite upshot is perceived for dusty flow. Figures 6, 7, 8, 9, 10, 11, 12 depict the outcome of sundry parameters on the temperature field of fluid and dusty granules i.e., $$\theta \left( \zeta  \right)$$ and $$\theta _{p} \left( \zeta  \right)$$. The outcome of the radiation parameter $$Rd$$ on $$\theta \left( \zeta  \right)$$ and $$\theta _{p} \left( \zeta  \right)$$ is discussed in Fig. 6a,b. Since $$Rd = \frac{{4\bar{\sigma }T_{\infty }^{3} }}{{3\bar{k}{\text{ }}k}},$$ so by up surging $$Rd$$ the mean absorption coefficient decreases. It is perceived that on escalating $$Rd$$ additional heat is produced in the system. Therefore, due to growing values of $$Rd$$ more heat is transmitted to the fluid. Hence, $$\theta \left( \zeta  \right)$$ and $$\theta _{p} \left( \zeta  \right)$$ rise for suspended particle and fluid phase. Figure 7a,b is sketched to analyze the behavior of heat transfer Biot number $$H_{1}$$ on $$\theta \left( \zeta  \right)$$ and $$\theta _{p} \left( \zeta  \right)$$. For growing values of $$H_{1}$$ heat transfer coefficient intensifies. On elevating $$H_{1}$$ fluid flow accelerates. Thus, $$\theta \left( \zeta  \right)$$ and $$\theta _{p} \left( \zeta  \right)$$ escalates on augmenting $$H_{1}$$. The performance of the thermal conductivity parameter $$d$$ on $$\theta \left( \zeta  \right)$$ and $$\theta _{p} \left( \zeta  \right)$$ is addressed in Fig. 8a,b. On accelerating $$d$$ temperature-dependent thermal conductivity amplifies. It is seen that rising values of $$d$$, results in an amplified collision among the particles. This leads to more exchange of heat through the fluid. Thus, $$\theta \left( \zeta  \right)$$ and $$\theta _{p} \left( \zeta  \right)$$ elevates on augmenting $$d$$ for both phases. Consequently, thicker penetration depth increases due to convective heat transfer at the surface. Figure 9a,b illustrate the fluctuation in fluid-particle interaction parameter $$\delta _{r}$$ for both phases $$\theta \left( \zeta  
\right)$$ and $$\theta _{p} \left( \zeta  \right)$$. It is witnessed that on incrementing $$\delta _{r}$$ fluid flow slows down. This corresponds to a decline in fluid flow. However, growing values of $$\delta _{r}$$ in suspended particles strengthen the frictional force. Hence, a reverse trend is observed for $$\theta _{p} \left( \zeta  \right)$$. The impact of thermophoresis parameter $$N_{t}$$ on $$\theta \left( \zeta  \right)$$ and $$\theta _{p} \left( \zeta  \right)$$ is displayed in Fig. 10a,b. It is observed that on enhancing $$N_{t}$$, thermophoretic force is strengthened. As a result, fluid particles move from hot to cold fluid. Thus, $$\theta \left( \zeta  \right)$$ and $$\theta _{p} \left( \zeta  \right)$$ augment. Figure 11a,b illustrate the impression of the Brownian motion parameter $$N_{b}$$ on $$\theta \left( \zeta  \right)$$ and $$\theta _{p} \left( \zeta  \right)$$. For growing values of $$N_{b}$$ collision among the fluid particles increases due to which more heat is generated. Therefore, $$\theta \left( \zeta  \right)$$ and $$\theta _{p} \left( \zeta  \right)$$ rises. To understand the variation of Dufour number $$D_{f}$$ on $$\theta \left( \zeta  \right)$$ and $$\theta _{p} \left( \zeta  \right)$$ Fig. 12a,b is plotted. On escalating $$D_{f}$$ concentration gradient enhances which results in heat transmission. Thus, a prominent upsurge is found in the thermal state of $$\theta \left( \zeta  \right)$$ and $$\theta _{p} \left( \zeta  \right)$$. The impression of varying Schmidt number $$S_{c}$$ on the concentration field $$\phi \left( \zeta  \right)$$ is discussed in Fig. 13. As $$S_{c}$$ is the quotient of kinematic viscosity $$v$$ to Brownian diffusion coefficient $$D_{B}$$. It is observed that rising values of $$S_{c}$$ diminishes the Brownian motion parameter. Thus, mass diffusion reduces for growing values of $$S_{c}$$. This results in the reduction of the concentration of the fluid. Therefore, deteriorating nature is exhibited by $$\phi \left( \zeta  \right)$$ on boosting $$S_{c}$$. Figure 14 is drawn to elucidate the upshot of dimensionless chemical reaction parameter $$\delta$$ on $$\phi \left( \zeta  \right)$$. On up surging $$\delta$$ chemical molecular diffusivity reduces owing to its consumption in the reaction. A slight decrement is observed in the boundary layer thickness. Thus, the concentration of the fluid deteriorates. The influence of variable molecular diffusivity $$e$$ on $$\phi \left( \zeta  \right)$$ is exhibited in Fig. 15. Since $$e$$ is proportionate to $$\phi \left( \zeta  \right)$$. For mounting values of $$e$$ variable mass diffusion elevates. Consequently, $$\phi \left( \zeta  \right)$$ augments. The impact of rising values of activation energy $$E$$ is deliberated in Fig. 16. It is noticed that escalating values of $$E$$ lead to a decrease in the Arrhenius function. Consequently, the generative chemical reaction decelerates. Thus, on accelerating $$E$$, the fluid concentration upsurges. Figure 17 is sketched to analyze the effect of Soret number $$S_{r}$$ on $$\phi \left( \zeta  \right)$$. $$S_{r}$$ is the quotient of difference in temperature and concentration. On escalating $$S_{r}$$, the temperature gradient rises. It is perceived that molecular diffusion increases. Thus, the rate of mass transfer intensifies for growing values of $$S_{r}$$. Consequently, $$\phi \left( \zeta  \right)$$ enhances.

The outcome of tabulated values of dimensionless parameters $\lambda _{1} ,H_{a} ,\delta ,$ and $L$ on drag force coefficient is depicted in Table 2. It is perceived that on escalating $\lambda _{1} ,H_{a} ,\,{\text{and}}\,\,\delta .$ shear stress increases. The influence of $\Pr ,Rd,D_{f} ,N_{b} ,S_{c} ,\delta ,S_{r} ,$ and $\delta _{T}$ on local Nusselt number and Sherwood number is portrayed in Table 3. It is perceived that on escalating $Rd$ heat and mass flux both augments. For growing values of $D_{f} ,N_{b} ,S_{c} ,$ and $\delta$ heat flux diminishes, whereas, mass flux upsurges. A deteriorating nature is exhibited by mass transfer on amplifying $S_{r}$ and $\delta _{r}$, however, the rate of heat transfer amplifies. A comparative analysis of the present investigation is exhibited in Table 4 with Wang^68^. A good association between the results is seen.

**Table 2:** Computational values of friction drag coefficient for distinct values of $\lambda _{1} ,Ha,L{\text{ }}\,{\text{and }}\,L.$

$\lambda _{1}$	$Ha$	$\delta _{v}$	$L$	$- \left( {\text{Re} _{x} } \right)^{{0.5}} C_{{f_{x} }}$	$- \left( {\text{Re} _{x} } \right)^{{0.5}} C_{{f_{y} }}$
0.5				1.0587562	0.10587562
0.6				1.0719469	0.10719469
0.7				1.0845045	0.10845045
	0.3			0.98104151	0.098104151
	0.4			0.99841054	0.099841055
	0.5			1.0147795	0.10147795
		0.3		1.0060463	0.10060463
		0.5		1.0147795	0.10147795
		0.7		1.0213022	0.10213022
			0.4	1.1423770	0.1142377
			0.5	1.0147795	0.10147795
			0.6	0.9139351	0.09139351

**Table 3:** Computational values of $Nu_{x} \text{Re} _{x}^{{ - 0.5}} {\text{ and }}Sh_{x} \text{Re} _{x}^{{ - 0.5}}$ against different estimation of $\Pr ,Rd,D_{f} ,N_{b} ,S_{c} ,\delta ,S_{r} {\text{ and }}\delta _{T} .$

$\Pr$	$Rd$	$D_{f}$	$N_{b}$	$S_{c}$	$\delta$	$S_{r}$	$\delta _{T}$	$Nu_{x} \left( {\text{Re} _{x} } \right)^{{ - 0.5}}$	$Sh_{x} \left( {\text{Re} _{x} } \right)^{{ - 0.5}}$
3								0.26287551	0.27159206
5								0.28311948	0.26845204
7								0.2946139	0.26679483
	0.3							0.24448878	0.27460726
	0.6							0.29656071	0.27649881
	0.9							0.34511853	0.27805908
		0.3						0.22529783	0.39278698
		0.5						0.20025387	0.39816084
		0.7						0.17477315	0.40337968
			0.2					0.24141581	0.27734281
			0.5					0.23190415	0.27863757
			0.7					0.2253281	0.27953848
				0.6				0.20919744	0.49599463
				0.8				0.19513655	0.58476006
				1.2				0.17110717	0.73964817
					0.4			0.23334648	0.34198783
					0.6			0.22529783	0.39540692
					0.8			0.21795961	0.44369725
						0.4		0.17026475	0.42250068
						0.6		0.17169455	0.41714266
						0.8		0.17319573	0.41215419
							0.3	0.22529789	0.42526637
							0.6	0.25082223	0.41921075
							0.9	0.26650763	0.41543192

**Table 4:** Comparison of $f''\left( 0 \right),j''\left( 0 \right),f\left( \infty  \right){\text{ and }}j\left( \infty  \right)$ for numeric values of $P$ with Wang^68^.

$P$	$f''\left( 0 \right)$		$j''\left( 0 \right)$		$f\left( \infty \right)$		$j\left( \infty \right)$	
	^68^	Present	^68^	Present	^68^	Present	^68^	Present
0	− 1	− 1	0	0	1	1	0	0
0.25	− 1.048813	− 1.048762	− 0.194564	− 0.194534	0.907075	0.907052	0.257986	0.257974
0.5	− 1.093097	− 1.093092	− 0.465205	− 0.465127	0.842360	0.842325	0.451671	0.451635
0.75	− 1.134485	− 1.134453	− 0.794622	− 0.794612	0.792308	0.792353	0.612049	0.612026
1	− 1.173720	− 1.173628	− 1.173720	− 1.173724	0.751527	0.751516	0.751527	0.751525

## Concluding remarks

Numerical solution for dusty radiative Casson nanofluid flow with temperature-dependent thermal conductivity and variable molecular mass diffusion has been investigated past a deformable bidirectional surface. Transfer of heat and mass is enhanced by inspecting the impression of the Soret–Dufour factor amalgamated with chemical reaction and activation energy. The flow is incorporated with additional effects of momentum slip and convective heat conditions. The mathematical model is deciphered through bvp4c, an implemented function in MATLAB. The perceptible analyses of the present exploration are:

- For growing values of $\beta ,\lambda _{1} ,$ and $L$ velocity field declines for fluid-particle suspension.
- A reverse trend is noticed in the velocity field for enhancing $\delta _{v}$ for both phases.
- An increasing behavior is exhibited by the thermal field for growing values of $Rd,H_{1} ,D_{f}$ and $N_{t}$ for fluid and dust phase.
- An opposite behavior is noticed in the thermal field for fluctuation in fluid-particle interaction parameters for the fluid and dust phase.
- For larger values of $S_{c} ,$ and $\delta ,$ the concentration field declines.
- The concentration field augments on amplifying $E$ and $S.$
- Drag force coefficient increases on escalating $\lambda _{1} ,H_{a} ,$ and $\delta _{v} .$
- The mass transfer exhibits a deteriorating impact on amplifying $S_{r} ,$ and $\delta _{r} ,$ however, the rate of heat transfer amplifies.
- Heat and mass flux augments on escalating $$Rd$$$$Rd$$.

## Nomenclature

$$b$$: Positive constant
$B_{0}$: Magnetic field strength
$$c$$: Positive constant
$$c_{s}$$: Concentration susceptibility
$c_{p}$: Specific heat capacity of the fluid
$C_{w}$: Concentration at the surface
$C_{\infty }$: Fluid ambient concentration
$c_{m}$: Specific heat of dust particle
$C_{f}$: Skin friction coefficient
$D_{T}$: Thermophoretic diffusion coefficient
$D_{B}$: Brownian diffusion coefficient
$D_{B} \left( C \right)$: Variable molecular diffusivity
$D_{{B_{\infty } }}$: Ambient diffusion coefficient
$d$: Variable thermal conductivity parameter
$$D_{f} = \frac{{D_{T} k_{t}^{*} \left( {C_{w} - C_{\infty } } \right)}}{{\nu c_{s} c_{p} \left( {T_{w} - T_{\infty } } \right)}}$$: Dufour number
$$E_{a}$$: Activation energy
$E = \frac{{E_{a} }}{{kT}}$: Activation energy parameter
$e$: Variable molecular diffusivity parameter
$h_{1}$: Convective heat transfer coefficient
$$Ha = \frac{{\sigma B_{o}^{2} }}{{\rho c}}$$: Hartmann number
$H_{1} = \frac{{h_{1} }}{{k_{\infty } }}\sqrt {\frac{\nu }{c}}$: Heat transfer Biot number
$K = 6\pi \mu r$: Stoke’s drag constant
$k(T)$: Temperature-dependent thermal conductivity
$\bar{k}$: Mean absorption coefficient
$K^{*}$: Permeability of porous medium
$k_{r}^{2}$: Chemical reaction parameter
$$k_{t}^{*}$$: Thermal diffusion
$L = S\sqrt {\frac{c}{\nu }}$: Velocity slip parameter
$m$: Mass of dust particle
$n$: Fitted rate constant
$N_{b} = \frac{{\tau \left( {C_{w} - C_{\infty } } \right)D_{B} }}{\nu }$: Brownian motion parameter
$$N_{t} = \frac{{\tau D_{T} \left( {T_{w} - T_{\infty } } \right)}}{{\nu T_{\infty } }}$$: Thermophoretic parameter
$Nu_{x}$: Local Nusselt number
$P = \frac{b}{c}$: Stretching ratio parameter
$$\Pr = \frac{{\mu c_{p} }}{{k_{\infty } }}$$: Prandtl number
$q_{r}$: Radiative heat flux
$$Q_{w}$$: Heat flux
$$Q_{m}$$: Mass flux
$\text{Re} = \frac{{c\left( {x + y} \right)^{2} }}{\nu }$: Local Reynold number
$$Rd = \frac{{4\bar{\sigma }T_{\infty }^{3} }}{{\bar{k}k_{\infty } }}$$: Radiation parameter
$r$: The radius of a dust particle
$S$: Velocity slip factor
$$S_{c} = \frac{\nu }{{D_{B} }}$$: Schmidt number
$$S_{r} = \frac{{D_{T} k_{t}^{*} \left( {T_{w} - T_{\infty } } \right)}}{{\nu T_{m} \left( {C_{w} - C_{\infty } } \right)}}$$: Soret number
$Sh_{x}$: Local Sherwood number
$T$: Temperature of fluid
$T_{p}$: The temperature of the dust particle
$T_{w}$: The temperature at the surface of a sheet
$T_{\infty }$: Fluid ambient temperature
$u,v,w$: Component of velocity
$u_{p} ,v_{p} ,w_{p}$: The velocity of dust particles
$$x,y,z$$: Cartesian coordinate

## Greek symbols

$$\rho$$: Fluid density
$\lambda = \frac{{Nm}}{\rho }$: Mass concentration of dusty granules
$$\sigma _{1}$$: Electrical conductivity
$$\beta$$: Casson parameter
$\tau$: The quotient of effective heat capacity of nanoparticle to the heat capacity of liquid
$\tau _{v} = \frac{m}{K}$: The relaxation time of the dust particle
$\rho _{p} = mN$: The density of dust particle
$\bar{\sigma }$: Stefan Boltzmann constant
$$\lambda _{1} = \frac{\nu }{{K^{*} c}}$$: Porosity parameter
$\delta _{v} = \frac{1}{{\tau _{v} c}}$: Fluid particle interaction parameter for velocity
$\tau _{T}$: Thermal equilibrium time
$$\nu$$: Kinematic viscosity
$$\zeta$$: Dimensionless variable
$$\alpha = \frac{{T_{w} - T_{\infty } }}{{T_{\infty } }}$$: Temperature difference
$\gamma = \frac{{c_{p} }}{{c_{m} }}$: The ratio of specific heat
$\delta = \frac{{k_{r}^{2} }}{c}$: Dimensionless reaction rate
$\tau _{{zx}}$: Shear stress in the x-direction
$\tau _{{zy}}$: Shear stress in the y-direction
